# Cytosine methylations in the promoter regions of genes involved in the cellular oxidation equilibrium pathways affect rice heat tolerance

**DOI:** 10.1186/s12864-020-06975-3

**Published:** 2020-08-14

**Authors:** Chao He, Hong-Yu Zhang, Yong-Xin Zhang, Pei Fu, Li-Li You, Wen-Bo Xiao, Zhao-Hai Wang, Hai-Yan Song, Ying-Jin Huang, Jiang-Lin Liao

**Affiliations:** 1 Key Laboratory of Crop Physiology, Ecology and Genetic Breeding (Jiangxi Agricultural University), Ministry of Education of China, Nanchang, 330045 China; 2 South Zhejiang Key Laboratory of Crop Breeding, Institute of Crop Research, Wenzhou Academy of Agricultural Sciences, Wenzhou, 325006 China; 3 Southern Regional Collaborative Innovation Center for Grain and Oil Crops in China, Changsha, 410128 China

**Keywords:** Rice, *Oryza sativa*, High temperature, Epigenetics, Cytosine methylation

## Abstract

**Background:**

High temperatures, particularly at night, decrease rice yield and quality. As high nighttime temperatures (HNTs) become increasingly frequent due to climate change, it is imperative to develop rice crops that tolerate HNTs. DNA methylation may represent a potential avenue for HNT-tolerant rice strain development, as this mechanism regulates gene activity and cellular phenotype in response to adverse environmental conditions without changing the nucleotide sequence.

**Results:**

After HNT exposure, the methylation patterns of cytosines in the CHH context differed noticeably between two coisogenic rice strains with significantly different levels in heat tolerance. Methylation differences between strains were primarily observed on successive cytosines in the promoter or downstream regions of transcription factors and transposon elements. In contrast to the heat-sensitive rice strain, the regions 358–359 bp and 2–60 bp downstream of two basal transcriptional factors (*TFIID subunit 11* and *mediator of RNA polymerase II transcription subunit 31*, respectively) were fully demethylated in the heat-tolerant strain after HNT exposure. In the heat-tolerant strain, HNTs reversed the methylation patterns of successive cytosines in the promoter regions of various genes involved in abscisic acid (ABA)-related reactive oxygen species (ROS) equilibrium pathways, including the pentatricopeptide repeat domain gene *PPR* (*LOC_Os07g28900*) and the homeobox domain gene *homeobox* (*LOC_Os01g19694*). Indeed, *PRR* expression was inhibited in heat-sensitive rice strains, and the methylation rates of the cytosines in the promoter region of *PRR* were greater in heat-sensitive strains as compared to heat-tolerant strains.

**Conclusions:**

After HNT exposure, cytosines in the CHH context were more likely than cytosines in other contexts to be methylated differently between the heat-sensitive and heat-tolerant rice strains. Methylation in the promoter regions of the genes associated with ABA-related oxidation and ROS scavenging improved heat tolerance in rice. Our results help to clarify the molecular mechanisms underlying rice heat tolerance.

## Background

The grain weight of rice (*Oryza sativa*), an important component of yield, is predominantly determined by the rate and duration of grain filling during the milky stage. However, at the milky stage, rice is extremely sensitive to high temperatures, especially high nighttime temperatures (HNTs). During the milky stage, HNTs are more harmful to rice grain yield than high daytime temperatures due to the increased dark respiration rates in the leaves and culms. Increased rates of dark respiration concomitantly increase respiration loss, leading to decreases in grain size, grain weight, and, ultimately, yield loss [1, 2].

High temperatures affect protein synthesis processes in rice grains (e.g., protein transport, folding, and assembly), and increase the glutamate, aspartate, asparagine, alanine, and sucrose contents of the grains, resulting in chalky grains [3, 4]. High temperature exposure suppresses of genes related to starch biosynthesis, including the *granule-bound starch synthase I* (*GBSSI*), *soluble starch synthase IIa* and *IIIa* (*SSSIIa* and *SSSIIIa*), *starch branching enzyme I* and *IIb* (*SBEI* and *SBEIIb*), *ADP-glucose pyrophosphorylase* (*AGPase*), and *cytosolic pyruvate orthophosphate dikinase* (*cyPPDK*) [5–8]. Thus, rice plants exposed to high temperatures produce grains containing large starch granules with medium-long starch chains but with low contents of amylose in the endosperm cells [9, 10]. High temperatures also induce the upregulation of genes associated with starch hydrolysis, including *starch debranching enzyme* (*DBE*) and α-amylase genes (*Amy1A*, *Amy1C*, *Amy3A*, *Amy3D*, and *Amy3E*), resulting in increased chalkiness and loosely-packed spherical starch granules in the rice grain [8, 11]. In plant cells, high temperature stress also triggers the expression of heat shock transcription factor (*Hsf*) genes [12] and NADPH oxidase-related genes, as well as genes associated with gibberellin (GA) and/or abscisic acid (ABA) catabolism, and the expression of these genes leads to reactive oxygen species (ROS) accumulation and chalky grains [13]. These previous studies have provided an indication of the molecular mechanisms and regulatory networks associated with rice quality that are affected by elevated temperatures during the grain-filling stage.

Epigenetic mechanisms are those that induce heritable changes in gene activity without altering the DNA sequence. Cytosine methylation in genomic DNA has long been considered as one of the most important epigenetic mechanisms in most common eukaryotic cells [14, 15]. Cytosine methylation in genomic DNA plays an important role in regulating the expression of coding and non-coding genetic elements at the transcriptional and post-transcriptional levels [16]. That is cytosine methylation enables the regulation of gene activity without changing the DNA sequences; this mechanism may thus represent an adaptive strategy that allows plants to survive and reproduce under adverse environmental conditions [16]. In *Arabidopsis*, the genome-wide inhibition of cytosine methylation might enhance heat tolerance; heat stress induced the cytosine methylation of the target gene or its adjacent transposon elements (TEs) to suppress target gene expression, thus leading to transcriptional reprogramming [17, 18]. In rice, the DNA methylation status of the gene *fertilization-independent endosperm1* (*OsFIE1*) may affect endosperm development during temperature adaption [19].

Previously, we reported that the genes involved in electron transport and oxidation equilibrium in plant cells were disrupted by short-term extreme HNTs, and these changes in hydrogen ion concentrations in the mitochondrial and cellular matrices, and these changes triggered and regulated downstream stress-response genes and proteins involved in photosynthesis, nucleotide catabolism, and the S-adenosylmethionine (SAM) cycle, resulting in abnormal grain-filling in rice [20–22]. However, the primary genetic elements regulating the gene and/or protein activity in the rice stress-response network after HNT exposure require further exploration.

**Fig. 1:**
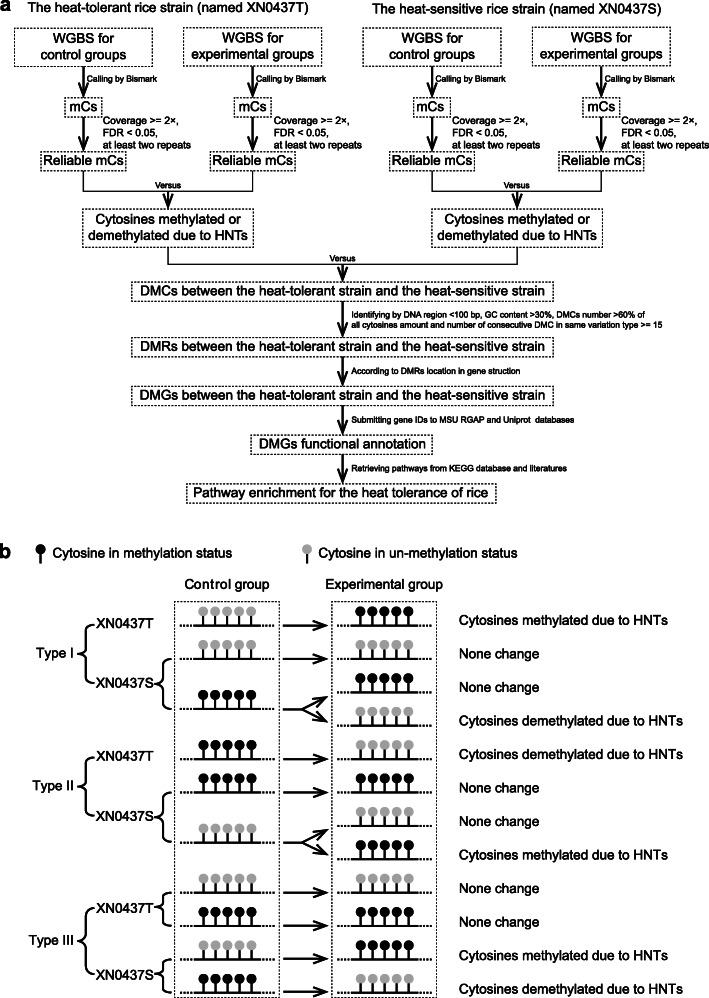
Overview of the methylation analysis workflow (**a**) and cytosine variation induced by high nighttime temperatures (**b**). WGBS, whole-genome bisulfite sequencing; HNT, high nighttime temperature; mC, methylated cytosine; DMC, a cytosine differentially methylated between the heat-tolerant and heat-sensitive coisogenic rice strains after exposure to HNTs; DMR, a DNA region < 100 bp long with a GC content of > 30%, where > 60% of all cytosines in the region were DMCs and at least 15 successive DMCs exhibited identical patterns of consistent methylation or demethylation; DMG, a differentially methylated gene adjacent to a DMR. Type I DMCs were methylated by HNTs in the heat-tolerant rice strain XN0437T, but the corresponding cytosine in the heat-sensitive rice strain XN0437S was either unchanged or demethylated after HNT. Type II DMCs were demethylated by HNTs in XN0437T, but the corresponding cytosine in XN0437S was either unchanged or methylated after HNT. Type III DMCs were unchanged by HNT in XN0437T, but the corresponding cytosine in XN0437S was either methylated or demethylated after HNT

Here, we performed whole-genome bisulfite sequencing (WGBS) to analyze differences in the patterns of cytosine methylation in response to short-term extreme HNT between two coisogenic rice strains with significantly different levels of heat tolerance (Fig. 1a). Our aim was to explore the primary genetic elements involved in rice heat tolerance.

## Results

### Two coisogenic rice strains were differently affected by HNT stress

**Fig. 2:**
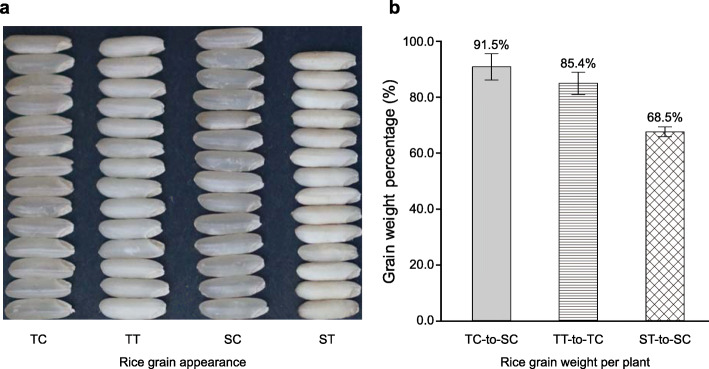
Differences in rice grain appearance (**a**) and grain weight (**b**) after high nighttime temperature (HNT) exposure. TC and TT indicate the heat-tolerant rice strain under control and HNT conditions, respectively; SC and ST indicate the heat-sensitive rice strain under control and HNT conditions, respectively; TC-to-SC indicates the difference in grain weight between TC and SC; TT-to-TC indicates the difference in grain weight between TT and TC; ST-to-SC indicates the difference in grain weight between ST and SC

HNT stress reduced the grain transparency of both the heat-tolerant and the heat-sensitive coisogenic rice strains. However, the HNT-exposed grains of the heat-sensitive strain were obviously smaller than those of the HNT-exposed heat-tolerant strain (Fig. 2a). Under control conditions, the grains of the heat-tolerant strain were 91.5% of the weight of the grains of the heat-sensitive strain (91.5%; *P* = 0.278; Fig. 2b). After exposure to HNT, the grains of the heat-sensitive strain weighed significantly less than those of the unexposed heat-sensitive strain (68.5%; *P* < 0.01; Fig. 2b). In contrast, although the grains of the HNT-exposed heat-tolerant strain were somewhat lighter than those of the unexposed heat-tolerant strains (85.4%), this difference was not significant (*P* = 0.069; Fig. 2b).

### Raw data and clean data

WGBS and base calling generated 689.26 million raw reads across all 12 samples, with 45.83–70.97 million reads per sample (Additional file 1: Table S1). After filtering, we obtained 595.55 million clean reads (86.4% of the raw reads) that were > 35 bp long, had an N content < 10%, and possessed a Phred quality score > 20.

### Genome-wide DNA methylation of cytosines

**Fig. 3:**
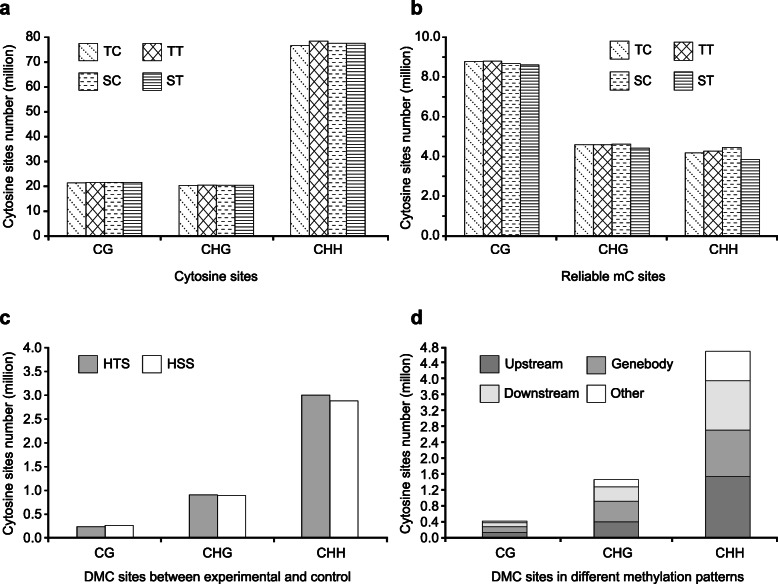
Number of CG, CHG, and CHH cytosine contexts in the two coisogenic rice strains. (**a**) Number of cytosines in the TC, TT, SC, and ST samples. (**b**) Number of reliable mCs in the TC, TT, SC, and ST samples. (**c**) Number of DMCs in the TC, TT, SC, and ST samples. (**d**) Number of DMCs that were differentially methylated between the heat-tolerant and heat-sensitive coisogenic rice strains. HTS, heat-tolerant strain; HSS, heat-sensitive strain; mC, methylated cytosine; TC and TT, heat-tolerant strain under control and high nighttime temperature conditions, respectively; SC and ST, heat-sensitive strain under control and high nighttime temperature conditions, respectively; DMC, a cytosine differentially methylated between the heat-tolerant and heat-sensitive coisogenic rice strains

Mapping of the bisulfite-converted clean reads to the bisulfite-converted reference genome yielded 284.60 million unique best-mapped reads across all 12 samples (Additional file 1: Table S1). The unique best-mapped reads for each sample covered 68.6–75.2% of the reference genome. By mapping these unique best-mapped reads to the reference genome without bisulfite conversion, we detected > 111.94 million cytosines per sample. Of these cytosines, an average of 21.61 million per sample were in the CG context (over 17.9% of all cytosines); an average of 20.36 million per sample (over 16.9%) were in the CHG context (where H represents A, C, or T); and an average of 77.76 million per sample (over 64.8%) were in the CHH context (Fig. 3a and Additional file 1: Table S2).

Methylated cytosine (mCs) calling identified 16.2–18.4% of the cytosines in each samples as mCs (Additional file 1: Table S2). All mCs that appeared in at least two replicates with the same methylation status, and which had a read coverage ≥2× and a false discovery rate (FDR) < 0.05, were considered reliable mCs. We obtained over 16.85 million reliable mCs per sample. Of the reliable mCs, 8.59–8.78 million (48.8–51.0%) were in the CG context, 4.42–4.61 million (26.1–26.2%) were in the CHG context, and 3.84–4.45 million (22.8–25.1%) were in the CHH context (Fig. 3b and Additional file 1: Table S2). Interestingly, most of the cytosines in the rice genome were in the CHH context, while few were in the CG and CHG contexts (Fig. 3a). In contrast, most of the mCs were in the CG context; few were in the CHG and CHH contexts (Fig. 3b).

### Differentially methylated cytosines between the experimental group and the control

In the heat-tolerant rice strain, we identified 4.14 million differentially methylated cytosines (DMCs) between the experimental group and the control. Of the DMCs, 2.13 million were methylated due to HNT and 2.01 million were demethylated. In the heat-sensitive rice strain, we identified 4.02 million DMCs between the experimental group and the control: 1.58 million were methylated due to HNT, and 2.44 million were demethylated (Fig. 3c).

### Different DMC methylation patterns in the two coisogenic rice strains

After identifying the DMCs between the experimental and control groups, we compared the methylation patterns of these DMCs between the heat-tolerant and the heat-sensitive rice strains. A total of 6.52 million DMCs showed different methylation patterns between the two coisogenic rice strains. These DMCs were then classified as type I, type II, or type III DMCs based on their methylation patterns (see the description of the criteria in the Materials and Methods). Of the 6.52 million DMCs, 1.90 million were type I DMCs, 1.69 million were type II DMCs, and 2.93 million were type III DMCs. Across all 6.52 million DMCs, the ratio of those in CG, CHG, and CHH contexts was approximately 1:3:11; most of the DMCs in the CG and CHG contexts were found in the gene body (34.8 and 35.7%, respectively). Conversely, most of the DMCs in the CHH context (32.5%) were found in the promoter region (Fig. 3d).

### Differentially methylated regions and its associated genes

**Fig. 4:**
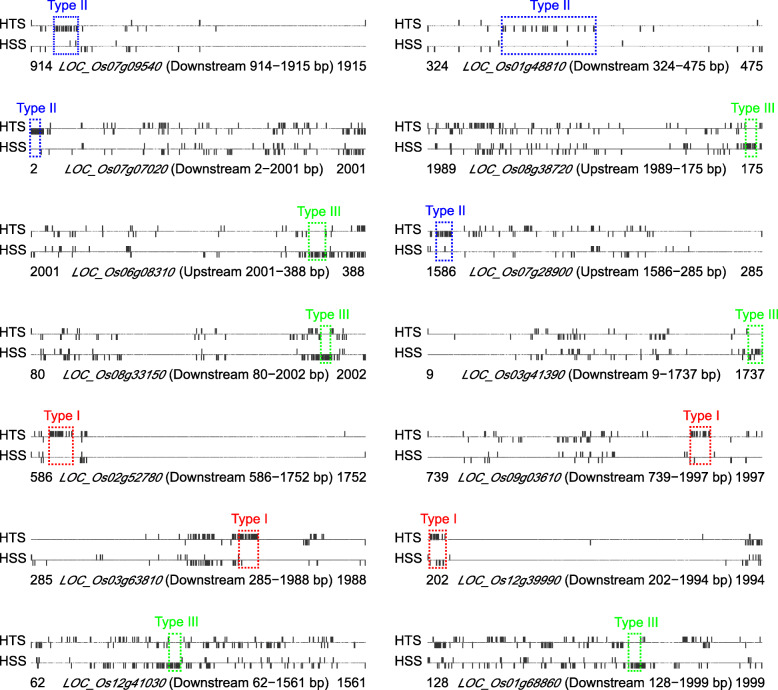
DMC types and locations in the DMRs of the target genes. DMC, a cytosine differentially methylated between the heat-tolerant and heat-sensitive coisogenic rice strains; DMR, a DNA region < 100 bp long with a GC content of > 30%, where > 60% of all cytosines in the region were DMCs and at least 15 successive DMCs exhibited identical patterns of consistent methylation or demethylation; HTS and HSS, heat-tolerant and heat-sensitive strain, respectively. Dots along the horizontal axes represent cytosines. Type I DMCs (in red) were methylated by HNTs in the heat-tolerant rice strain XN0437T, but the corresponding cytosine in the heat-sensitive rice strain XN0437S was either unchanged or demethylated after HNT. Type II DMCs (in blue) were demethylated by HNTs in XN0437T, but the corresponding cytosine in XN0437S was either unchanged or methylated after HNT. Type III DMCs (in green) were unchanged by HNT in XN0437T, but the corresponding cytosine in XN0437S was either methylated or demethylated after HNT. Vertical bars extending above the horizontal axes represent cytosines that were methylated by HNTs, while vertical bars extending below the horizontal axes represent cytosines that were demethylated by HNTs. Red, blue, and green dashed-line boxes indicate respectively the type I, II, and III DMRs in the target genes

As the efficient initiation of gene transcription requires a nonmethylated DNA region downstream and/or upstream of the gene [23], we identified differentially methylated regions (DMRs) between the heat-tolerant and heat-sensitive coisogenic rice strains. A DMR was defined as a fragment within which the DMCs were consistently methylated or demethylated in consecutive cytosine regions, and where this pattern of methylation or demethylation differed between the heat-tolerant and heat-sensitive strains. In total, we detected 99 DMRs between the two coisogenic rice strains. The DMRs were also classified into three types based on the methylation patterns of the DMCs they encompassed. Of the 99 DMRs, 32 were type I DMCs, 27 were type II, and 40 were type III (Additional file 1: Table S3). Each of these DMRs was respectively located on the promoter, downstream, or gene body of its target gene: 47 DMRs were in the upstream promoter region of the target gene, 51 were downstream, and only one was in the gene body. The genes associated with the DMRs were considered HNT-induced differentially methylated genes (DMGs) between the heat-tolerant and heat-sensitive coisogenic rice strains. Of the DMGs, 14 were potentially important for the plant response to HNTs. The locations of the DMRs on these 14 DMGs are shown in Fig. 4; DMRs are mapped to the remaining 53 DMGs in Additional file 1: Figure S1.

DMRs validation using bisulfite-sequencing PCR (BSP) showed that the DMRs identified using BSP were basically consistent with the results of the WGBS (Additional file 1: Figure S2). That is, for a given cytosine, the ratio of methylated reads to total covered reads in the WGBS results was nearly equivalent to the ratio of methylated clones to total clones in the BSP results. For example, the results of the WGBS showed that more than 57% of the cytosines at 1924–1949 bp downstream of the transcription start site (TSS) of *LOC_Os04g02030* were methylated in the heat-tolerant rice strain under normal conditions; similarly, BSP indicated that in six of the 10 clones (60%), all cytosines in this region were methylated.

### Function of the differential methylation genes

Using the Michigan State University Rice Genome Annotation Project (MSU RGAP, http://rice.plantbiology.msu.edu) and UniProt databases (https://www.uniprot.org), we annotated the functions of 67 DMGs; the functions of an additional 32 DMGs were unknown (Additional file 1: Table S3). Based on these annotations, the 67 DMGs were classified into six functional groups: transcriptional regulation (32 genes; 32.3% of all DMGs), energy metabolism (10 genes; 10.1% of all DMGs), transport (eight genes; 8.1% of all DMGs), signal transduction (eight genes; 8.1% of all DMGs), metabolism (six genes; 6.1% of all DMGs), and oxidation (three genes; 3.0% of all DMGs). Almost half of the functionally annotated DMGs (32 DMGs; 47.8%) were associated with the regulation of gene transcription. Of these DMGs, 10 encoded transposons or retrotransposons, and 22 encoded transcription factors (TFs).

Based on the different methylation patterns of the different DMC types in the DMRs, two genes (*LOC_Os10g42460* and *LOC_Os07g41530*), each of which encoded a Ty3-gypsy subclass retrotransposon protein*,* were identified as type I DMCs in the gene DMR (Additional file 1: Table S3 and Figure S1). Two other genes (*LOC_Os01g48810* and *LOC_Os07g07020*) were identified as type II DMCs, and were located in the region downstream of the target gene (Fig. 4 and Additional file 1: Table S3). *LOC_Os01g48810* and *LOC_Os07g07020* respectively encoded a TFIID subunit 11 and a RNA polymerase II transcription subunit 31 mediator, which are critical to the initial process of gene transcription by RNA polymerase II [24].

### Pathways enriched in the DMGs

**Fig. 5:**
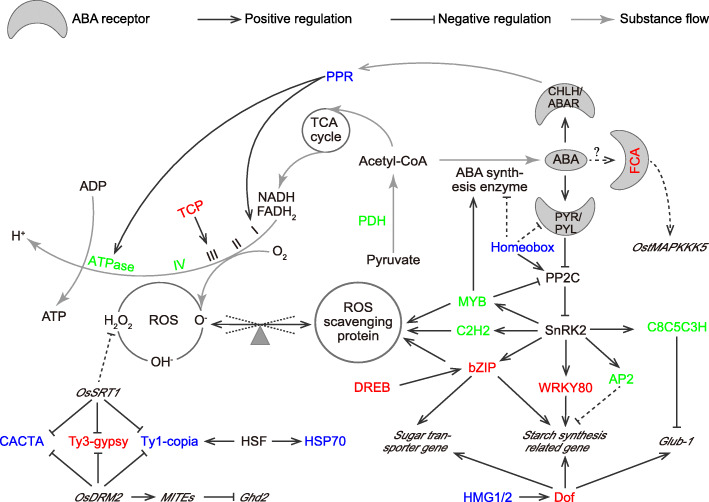
The regulatory network associated with the differentially methylated genes (DMGs). Type I DMGs are shown in red, type II DMGs are shown in blue, and type III DMGs are shown in green. PPR, transcription factor (TF) *pentatricopeptide repeat*; CHLH/ABAR, mg-chelatase H subunit/putative abscisic acid receptor; TCA, tricarboxylic acid cycle; Acetyl-CoA, acetyl coenzyme A; ABA, abscisic acid; FCA, *flowering control locus A*; TCP, TF *teosinte branched 1, cycloidea, pcf 1*; I, NADH dehydrogenase:ubiquinone oxidoreductase; II, succinate:ubiquinone oxidoreductase; III, cytochrome bc1 complex; IV, cytochrome c oxidase; PDH, pyruvate dehydrogenase; PYR/PYL, pyrabactin resistance/pyrabactin resistance-like ABA receptor; PP2C, phosphatases type 2C; ROS, reactive oxygen species; SnPK2, sucrose nonfermenting 1-related protein kinase2; MYB, TF *myeloblastosis*; C2H2, TF *cysteine 2/histidine 2 -type zinc finger proteins*; bZIP, TF *basic leucine zipper*; WRKY80, *WRKY family transcription factor 80*; AP2, TF *APETALA2*; C8C5C3H, TF *cysteine 8-cysteine 5 or cysteine 3-histidine-type zinc finger proteins*; CACTA, *En/Spm sub-class CACTA transposon protein*; Ty3-gypsy, *Ty3-gypsy* subclass retrotransposon protein; Ty1-copia, *Ty1-copia* subclass retrotransposon protein; HSF, *heat shock factor*; HSP70, *heat shock protein 70*; MITE, *miniature inverted repeat transposable element*; HMG1/2, TF *high mobility group 1/2*; Dof, TF *DNA binding with one finger protein*

Our composite regulatory network, which was based on the pathways enriched in the DMGs suggested that HNTs caused oxidation disequilibrium primarily by regulating ABA signaling pathways, and induced DNA methylation or demethylation in the genes involved in the oxidation disequilibrium process. The HNT-induced methylation of some genes, including *basic leucine zipper* (*bZIP*) [25], *dehydration-responsive element-binding protein* (*DREB*) [26] and *teosinte branched 1, cycloidea, pcf 1* (*TCP*) [27, 28], in conjunction with the demethylation of others, including *pentatricopeptide repeat* (*PPR*) [29, 30], and *homeobox* [31], might disrupt the normal regulation of oxidation disequilibrium by ABA in plant cells (Fig. 5).

### Differences in cytosine methylated rate, gene expression and grain weight among the six rice germplasms

**Fig. 6:**
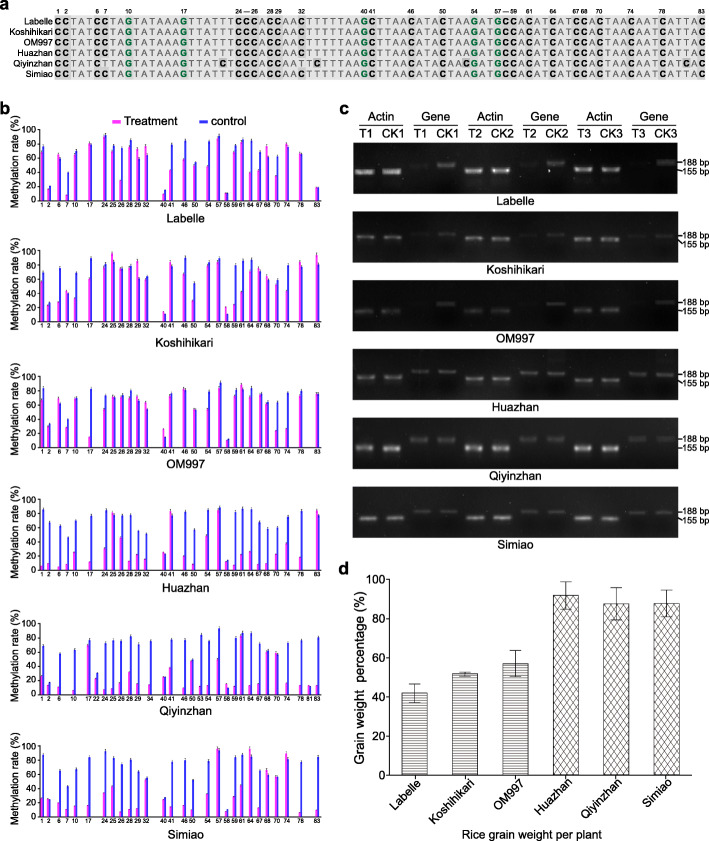
The DNA sequence, methylation ratio, expression patterns of gene PPR in six rice germplasms. (**a**) The nucleotide sequence 1495–1577 bp upstream from the TSS of gene *PPR* in the typical heat-sensitive (Labelle, Kashihikari and OM997) and heat-tolerant (Huazhan, Qiyinzhan and Simiao) rice germplasms. (**b**) The methylation rates of the cytosines 1495–1577 bp upstream of gene *PPR* in the heat-sensitive and heat-tolerant rice germplasms under high nighttime temperature and normal temperature conditions. (**c**) The gene expression patterns of *PPR* in the typical heat-sensitive and heat-tolerant rice germplasms under high nighttime temperature and normal temperature conditions (original full length gel images are presented in Additional file 2: Figure S4–S9); the rice *Actin1* (*LOC_Os03g50885*) gene was used as an internal control. T1, T2, and T3 indicate replicates exposed to high nighttime temperatures; CK1, CK2, and CK3 indicate control replicates kept at normal temperatures. (**d**) Weights of the rice grains produced by heat-sensitive and heat-tolerant germplasms exposed to either high nighttime temperatures or normal temperatures

In the heat-tolerant strain, the promoter regions of PPR family proteins, which have been identified as site-specific RNA editing factors [32], were demethylated by HNTs. In our regulatory network, these proteins were not only involved in the ABA signaling pathway, but also regulated oxidative phosphorylation (OXPHOS). Therefore, we compared heat tolerance, *PPR* cytosine methylation, and *PPR* gene expression patterns between heat-tolerant rice germplasms (Huazhan, Qiyinzhan, and Simiao) and heat-tolerant rice germplasms (Labelle, Koshihikari, and OM997). In the DNA sequence 1577–1495 bp upstream of *PPR*, we identified 28 cytosine sites in the rice germplasms Labelle, Koshihikari, OM997, Huazhan, and Simiao, and 30 cytosine sites in Qiyinzhan (Fig. 6a). The cytosine methylation rate (Fig. 6b) was similar across the six germplasms under normal temperature conditions. In general, the methylation rate was lower for the samples exposed to HNTs as compared to samples under normal temperature conditions. However, the methylation rates for the heat-tolerant rice germplasms (Huazhan, Qiyinzhan, and Simiao) were lower than those of the heat-sensitive germplasms (Labelle, Koshihikari and OM997). The agarose gel electrophoresis results (Fig. 6c) clearly indicated that the target gene (*PPR*) was suppressed in the heat-sensitive rice germplasms under HNT, as compared to under normal temperature conditions. In contrast, *PPR* gene expression in the heat-tolerant germplasms was similar between HNT and normal temperature conditions. The differences in grain weight between the HNT-exposed and control plants (Fig. 6d) were substantially greater for the heat-sensitive germplasms (Labelle, Koshihikari, and OM997) than for the heat-tolerant germplasms (Huazhan, Qiyinzhan, and Simiao).

## Discussion

Climate change-associated increases in temperature, particularly during the night, are detrimental to rice production and yield, as over-warm nighttime temperatures increase the rate of grain growth and decrease grain growth duration [33, 34]. The molecular mechanisms associated with the response of rice to HNT have been widely investigated over the past decade, and several of the genes, proteins, metabolites, and probable gene-regulatory networks involved in the rice HNT response have been explored [20–22]. However, the primary genetic elements and specific molecular mechanisms conferring rice heat tolerance remain unclear. Cytosine methylation in genomic DNA is an important epigenetic mechanism that may regulate the expression of coding and non-coding genetic elements at the transcriptional and post-transcriptional levels in response to adverse environmental conditions, without changing the DNA sequence [14, 16]. In *Arabidopsis*, cytosine methylation is involved in the regulation of gene expression, and participates in the response to temperature stress [18]. In rice, it was previously shown that the DNA methylation status of the gene *OsFIE1* was sensitive to temperature change and affected endosperm development [19]. Here, we demonstrated that HNT induced the demethylation of successive cytosines in the promoter regions of genes involved in ABA-related oxidation and ROS scavenging, thus contributing to rice heat tolerance.

In plants, cytosine methylation may occur in CG, CHG, or CHH contexts. CHH is the primary context, while CG is the primary context of mCs [16, 35]. However, in rice, variations in methylation status in response to stress and during growth are most common in the CHH context [36, 37]. Consistent with this, in both the heat-tolerant and the heat-sensitive coisogenic rice strains, over 64.8% of all cytosines were in the CHH context and 48.8–51.0% of all mCs were in the CG context. In contrast, most of the HNT-induced DMCs between the heat-tolerant and heat-sensitive coisogenic rice strains were identified in the CHH context. The results suggested that the methylation or demethylation of cytosine in the rice genome mostly occurs in the CG context, while CHH was the main context associated with HNT stress.

In plants, stress-activated TEs may rearrange gene expression networks to survive [38]. TEs are targeted by the de novo methylation machinery, which is controlled by methyltransferase DOMAINS REARRANGED METHYLTRANSFERASE 2 (DRM2) via RNA-directed DNA methylation (RdDM) [14]. For example, in rice, *OsDRM2* is negatively regulated by *micro RNA (miR) 820,* which is located within the *CACTA* TE [39]. In rapeseed, a CACTA-like transposable element in the upstream region of the gene encoding P450 monooxygenase acts as an enhancer, increasing silique length and seed weight [40]. In maize, the insertion of a CACTA-like TE into the promoter region of the photoperiod sensitivity modulator *ZmCCT* attenuates photoperiod sensitivity and accelerates postdomestication [41]. In addition, the expression of *GRMZM2G053177*, a candidate maize gene that may participate in the regulation of flavonoid pigment production (e.g., phlobaphenes), is controlled by the DNA methylation status of the inserted CACTA TE [42]. Our results showed that 18 successive cytosines in the region downstream of the CACTA TE (*LOC_Os07g09540*) were consistently demethylated by HNTs in the heat-tolerant rice strain (Fig. 4 and Additional file 1: Table S3). This suggested that the demethylation of the successive cytosines in the region downstream of the gene *LOC_Os07g09540* improved heat tolerance in rice.

During the initiative transcription of nearly all mRNAs, RNA polymerase II assembles with basal TFs, including TFIID and the mediator of RNA polymerase II, to form the transcription preinitiation complex (PIC) [43]. TFIID is a large, multi-subunit complex comprised of the TATA-binding protein (TBP) and TBP-associated factors (TAFs), which contain subunits capable of promoter DNA recognition. Mediator of RNA polymerase II acts as a checkpoint for gene activation and TFIID activity, and functions by relaying signals from TFs directly to RNA polymerase II, thereby facilitating the TF-dependent regulation of gene expression. Mediator of RNA polymerase II is also targeted by sequence-specific, DNA-binding TFs that work to control gene expression in response to developmental or environmental cues [44]. In the present study, we discovered 16 successive cytosines located 358–399 bp downstream of the transcription termination site (TTS) of the transcription initiation factor *TFIID subunit 11* (*LOC_Os01g48810*), that were consistently demethylated by HNTs in the heat-tolerant strain (Fig. 4 and Additional file 1: Table S3). In addition, 18 successive cytosines located 2–60 bp downstream of the TTS of the mediator of RNA polymerase II transcription *subunit 31* (*LOC_Os07g07020*) were also consistently demethylated by HNTs in the heat-tolerant rice strain (Fig. 4 and Additional file 1: Table S3). These results indicated that the mediator of RNA polymerase II regulates gene expression in response to developmental or environmental cues not only when targeted by sequence-specific DNA-binding TFs, but also as a result of the demethylation of the successive cytosines in the promoter regions of TFIID and the mediator of RNA polymerase II themselves [44].

Stress leads to high levels of ROS accumulation in plant cells, which may result in metabolic disorders, cellular damage, and premature senescence [45]. During cellular respiration, most ROS are generated by OXPHOS processes, which are performed by a set of membrane-associated enzymes and complexes, including complex I (NADH dehydrogenase ubiquinone oxidoreductase), complex II (succinate ubiquinone oxidoreductase), complex III (cytochrome bc1), complex IV (cytochrome c oxidase), and complex V (ATP synthase) [46]. Complex IV catalyzes water synthesis from hydrogen ions (H^+^) and oxygen (O^−^) to remove ROS, while complex V modulates ROS formation [46]. The inhibition of complex V leads to ROS accumulation during the OXPHOS process [47]. The activity levels of complex IV may be influenced by the DNA methylation of its components; for example, complex IV activity is inhibited by *COX7A1* methylation [48]. In addition, the expression levels of the components of complex V are influenced by the methylation of mitochondrial DNA [49]. Here, in the heat-tolerant rice strain, HNT did not affect the methylation statuses of 18 successive cytosines 209–260 bp upstream from the TSS of cytochrome C oxidase assembly protein *COX15* (*LOC_Os08g38720*), one component of complex IV. However, in the heat-sensitive strain, the corresponding cytosines were consistently methylated by HNTs. We also identified 19 successive cytosine, located 538–616 bp upstream from the TSS of the plasma membrane *H + -ATPase* (*LOC_Os06g08310*), that were consistently demethylated by HNTs in the heat-sensitive rice strain, but unaffected by HNTs in the heat-tolerant strain (Fig. 4 and Additional file 1: Table S3). These results indicated that, in rice, OXPHOS processes and ROS production might be disrupted by cytosine methylations in the promoter regions of *COX15* in complex IV and of *H*^*+*^*-ATPase* in complex V. These methylation events decrease heat tolerance in rice.

ABA is an important messenger that acts as a signaling mediator during oxidation and stress adaption. ABA reduces ROS damage in two ways: by inhibiting ROS production during OXPHOS, and by regulating the expression of genes related to ROS scavenging [50]. During OXPHOS, ABA binds to the receptor Mg-chelatase H subunit/putative ABA receptor (CHLH/ABAR) to induce TF *PPR*. *PPR* then regulates components of complex I and complex V post-transcription [30]. To influence the ROS scavenging pathway, ABA binds to the receptor pyrabactin resistance/pyrabactin resistance-Like (PYR/PYL) to inhibit downstream protein phosphatases type 2C (PP2C) [51]. PP2C inhibition indirectly activates the sucrose nonfermenting 1-related kinase2 (SnRK2), which then induces several downstream TFs, including *MYB*, *cysteine 2/histidine 2 -type zinc finger protein* (*C2H2),* and *bZIP* [25, 51–53]. Previous studies have shown that, in response to environmental stress or during development, DNA methylation in rice and *Arabidopsis* regulates *PPR* expression during OXPHOS, as well as *MYB*, *C2H2*, and *bZIP* in the ROS scavenging pathway [18, 54–56]. In the present study, we found that 21 successive cytosines, located 1495–1577 bp upstream from the TSS of the TF *PPR repeat domain containing protein* (*LOC_Os07g28900*), were consistently demethylated by HNTs in the heat-tolerant rice strain, whereas the corresponding cytosines in the heat-sensitive strain were unchanged or methylated by HNTs. In addition, 18 successive type III cytosines 1748–1799 bp downstream from the TTS of the MYB-family TF (*LOC_Os08g33150*), and 18 successive cytosines 1669–1737 bp downstream from the TTS of the TF *ZOS3–15-C2H2 zinc finger protein* (*LOC_Os03g41390*), were unchanged in response to HNTs in the heat-tolerant rice strain, whereas the corresponding cytosines in the heat-sensitive strain were either methylated or demethylated by HNTs. Finally, 20 successive cytosines, 651–728 bp downstream from the TTS of the TF *bZIP* (*LOC_Os02g52780*), were consistently methylated by HNTs in the heat-tolerant strain, but unaffected by HNTs in the heat-sensitive strain. Thus, the consistent methylation of successive cytosines in the promoter regions of various TFs might regulate ABA cellular oxidation pathways during the HNT response in rice (Fig. 4 and Additional file 1: Table S3). The consistent demethylation of the cytosines in the promoter region of *PPR*, the consistent methylation of the cytosines in the region downstream of *bZIP*, and the stable methylation states of the regions downstream of *MYB* and *C2H2* all improve heat tolerance in rice [18].

Several additional TFs, including flowering control locus A (*FCA*), *bZIP*, tryptophan-arginine-lysine-tyrosine (*WRKY*), APETALA2 (*AP2*), *cysteine 8-cysteine 5 or cysteine 3-histidine-type zinc finger protein* (*C8C5C3H*), and *DNA binding with one finger protein* (*Dof*), were also reported to be regulated by DNA methylation in plants [55–57]. The post-transcriptional regulator *FCA*, which has two RNA-binding motifs (RRMs), upregulates the gene mitogen-activated protein kinase 5 lacking an intact kinase domain (*OstMAPKKK5)*. The upregulation of *OstMAPKKK5* then increases endogenous GA content to affect the size of rice grain cells [58]. ABA-induced *bZIP* directly stimulates amylase- and sugar-transporter genes to increase the accumulation of soluble sugars in plants [59]. *WRKY* is involved in carbohydrate anabolism during starch synthesis in the endosperms of rice and wheat, while *AP2* regulates both embryo cell number and cell size by controlling sugar metabolism [60, 61]. *C8C5C3H* is associated with the regulation of the gene *Glub-1* to control glutelin accumulation in rice grains, and *Dof* influences the expression of seed-storage genes, including sugar transporter genes, starch synthesis genes, and glutelin genes [62, 63]. Here, we found that the successive cytosines in the promoter regions or the regions downstream of the TTS of *FCA* (*LOC_Os09g03610*), *bZIP* (*LOC_Os02g52780*), *WRKY80* (*LOC_Os03g63810*), *Dof* (*LOC_Os12g39990*), *AP2* (*LOC_Os12g41030*), and *C8C5C3H* (*LOC_Os01g68860*) were differentially methylated or demethylated between the heat-tolerant and heat-sensitive coisogenic rice strains after HNT exposure (Fig. 4 and Additional file 1: Table S3). Thus, the methylation statuses of the cytosines in the promoters or downstream regions of these TFs were associated with grain size, energy synthesis, and energy transport in rice, all of which ultimately affected heat tolerance.

Based on the DMG-associated pathways identified in the Kyoto Encyclopedia of Genes and Genomes (KEGG, https://www.genome.jp/kegg/genes.html) and in the literature, we constructed a composite DMG regulatory network (Fig. 5). In the constructed network, the successive cytosines in the region upstream of the gene encoding the TF *PPR*, which is activated when ABA binds to CHLH/ABAR and which participates in the regulation of complex I and complex V components [30], were consistently demethylated by HNTs. These cytosines also triggered cellular oxidation and ROS production in response to HNT stress. In contrast, the successive cytosines in the gene body of the TF *homeobox* were consistently demethylated by HNTs; these cytosines regulated the binding of ABA to PYR/PYL and activated PP2C. SnRK2, a key regulator of ROS scavengers (e.g., *MYB*, *C2H2*, and *bZIP*), is negatively regulated by PP2C [25, 51–53]. However, the cytosines in the promoter or downstream regions of the TFs *MYB* and *C2H2* were demethylated by HNTs in the heat-sensitive rice strain, but were unaffected by HNTs in the heat-tolerant strain. This methylation might permit the removal of excessive ROS and thus reduce cellular damage in the heat-tolerant strain. *SnRK2* also plays important roles in sugar transport and starch synthesis by regulating *bZIP*, *WRKY80*, *AP2,* and *C8C5C3H* [59–62]*.* The cytosines in the promoter or downstream regions of *bZIP* and *WRKY80* were methylated by HNTs in the heat-tolerant rice strain, while the cytosines in the promoter or downstream regions of *AP2* and *C8C5C3H* were unchanged. To confirm the relationship between ABA-related oxidation induction and ROS scavenging in plant cells under HNT stress, we measured the activity levels of ROS-related enzymes, including superoxide dismutase (SOD), peroxidase (POD) and catalase (CAT), as well as endogenous ABA concentrations, in the grains produced by the heat-tolerant and heat-sensitive rice germplasms. We found that, after HNT exposure, the activity levels of SOD, POD and CAT, as well as the endogenous ABA concentrations, were higher in the heat-tolerant rice grains than in the heat-sensitive rice grains (Additional file 1: Figure S3). Because the successive cytosines in the region upstream of *PPR* were demethylated by HNTs (Fig. 6b), and because ABA-related oxidation induction and ROS scavenging were vigorous in the heat-tolerant rice germplasms, this suggested that the demethylation of *PPR* regulated the downstream oxidation equilibrium via ABA-related oxidation induction and ROS scavenging, and that this demethylation improved heat tolerance in rice [21].

## Conclusions

The CHH context was the most common context of the mCs, and this context was differentially methylated between the heat-tolerant and the heat-sensitive rice strains in response to HNTs. The methylation or demethylation of successive cytosines in the promoter or downstream regions of the genes involved in ABA-related oxidation induction and ROS scavenging (e.g., methylations near *bZIP*, *DREB* and *TCP*, and demethylations near *PPR* and *homeobox*) improved rice heat tolerance. To our knowledge, this is the first report that successive cytosines in the promoter or downstream gene regions are consistently methylated or demethylated by HNTs, affecting cellular oxidation and heat tolerance in rice. However, several key questions remain unanswered, including how and why HNTs induce the methylation or demethylation of successive cytosines in the promoter or downstream region of these genes. In addition, the role played by gene activity in the consistent methylation or demethylation of successive cytosines in gene promoter regions in response to HNT stress remains unclear.

## Methods

### Plant materials

Two coisogenic rice strains with differing levels of heat tolerance (heat-tolerant and heat-sensitive) at the milky stage were used for WGBS analysis. Both rice strains were developed from recombinant inbred populations, the genomic polymorphism of which was only 1.80% based on 887 simple sequence repeat (SSR) markers. The grain weights of these strains differed significantly following HNT exposure at the milky stage [64].

### Rice cultivation, temperature treatment, and sampling

Rice was cultured following the tub-planting method described in previous studies [64]. To ensure that only uniformly-developed samples were used for genomic cytosine DNA methylation analysis, only rice ears with the same heading date and the same flowering date were selected and labeled. On the 8th day after the labeled florets flowered, the rice plants were moved into phytotrons at Jiangxi Agricultural University, China (28°75′ N, 115°83′ E) and heat shocked at 38.0 ± 0.5 °C during the dark period (10 h; 19:00–05:00). Control plants were kept at 25.0 ± 0.5 °C during the dark period. Control and experimental plants were maintained at 26 ± 0.5 °C during the light period (14 h; 5:00–19:00). After two dark and two light treatment periods (48 h in total), the rice plants were removed from the phytotrons. Then, 3 g of labeled caryopses (about 100 caryopses) from each plant were harvested, wrapped in tinfoil, snap-frozen in liquid nitrogen, and stored at − 80 °C for further analysis. Temperature treatments were performed on 12 plants with three biological replicates: heat-tolerant controls (TC1, TC2, and TC3), heat-tolerant experimental (TT1, TT2, and TT3), heat-sensitive controls (SC1, SC2, and SC3), and heat-sensitive experimental (ST1, ST2, and ST3).

### Grain weights

To compare grain weights between the rice strains subjected to HNT and the controls, labeled but un-sampled rice plants were removed from the phytotrons and allowed to continue to grow naturally. After the rice had matured, grains from five mature, labeled rice ears were randomly selected, harvested, dried, and weighed. The percentage differences in grain weight between the HNT-exposed plants and the control plants were then calculated for each strain.

### DNA library construction and whole-genome bisulfite sequencing

Genomic DNA was extracted from 0.5 g of the harvested caryopses from each sample. The quality and quantity of the extracted DNA were measured using 1% agarose gel electrophoresis and a Qubit Fluorimeter (Life Technologies, USA), respectively. We then sonicated 600 ng of each high-quality genomic DNA sample to generate approximate 400 bp DNA fragments. The DNA fragments were purified with 1.8 × Agencourt AMPure XP magnetic beads (Beckman Coulter, USA). Following end-repair, 3′-end adenylate and Illumina sequencing adapters were ligated using the NEXTflex Bisulfite-Seq Kit (BioO Scientific, USA), following the manufacturer’s instructions. The adapter-ligated DNA fragments were separated using 2% low-melting-point agarose gel electrophoresis. Target DNA fragments (400–500 bp long) were isolated from the gel and purified using the MinElute Gel Extraction Kits (Qiagen, Germany).

The purified DNA fragments were subjected to bisulfite treatment to convert each nonmethylated cytosine into a uracil (U) using the EZ DNA Methylation-Gold kit (Zymo, USA), following the manufacturer’s instructions. The bisulfite-treated DNA fragments were amplified using 14 cycles of PCR to enrich the bisulfite-treated DNA fragments; this process also transformed each U (nonmethylated cytosine) into a thymine (T). Thus, each nonmethylated cytosine was converted to U and then read as T when sequenced; mCs, which are protected from conversion, were still read as cytosines [65]. The libraries were run on a LabChip GX Touch (Perkin-Elmer, USA) to assess the size distribution of the library fragments, and the library concentration was quantified using a Qubit 2.0 Fluorimeter (Life Technologies, USA). Real-time quantitative (RTq) PCRs were performed using a KAPA Illumina Library Quantification kit (Kapa Biosystems, USA).

Qualified libraries were paired-end sequenced (2 × 150 bp) on a HiSeq 2500 (Illumina, USA). The peak signal generated by the Illumina HiSeq platform was used for base calling and was transformed into the base sequence as raw reads.

### Raw data filtration and reliable methylated cytosine calling

To avoid incorrect methylation calls due to low-quality reads, raw reads were filtered using Trim_Galore v0.4.2 (http://www.bioinformatics.babraham.ac.uk/projects/trim_galore/) to remove reads with adapter sequences, reads that were shorter than 35 bp, reads with more than 10% Ns, or reads with more than 50% low-quality bases (Phred quality score < 20).

To call mCs, the reference genome was first transformed into a bisulfite-converted version (i.e., cytosine-to-thymine and guanine-to-adenine conversions were performed) and then indexed using bowtie2 [66, 67]. The bisulfite-treated filtered reads were also transformed into fully bisulfite-converted versions (i.e., cytosine-to-thymine and guanine-to-adenine conversions were performed) and aligned to similarly-converted versions of the reference genome using Bismark v0.12.5 in a directional manner (parameters: -*N* = 1, −L = 20, −*p* = 5, −I = 0, −X = 500) [68]. The unique best-aligned reads were then compared to the unconverted reference genome to determine the methylation status of each cytosine (i.e., methylation or nonmethylation). If a site was a cytosine both in the unique best-aligned reads and in the reference genome, this cytosine in the unique reads was called as a mC. The data, including the number of mapped reads, cytosines, cytosine contexts (CG, CHG, or CHH), and cytosine methylation statuses in the reference genome, were retrieved by Bismark for further analysis. To ensure the reliability of the detected mCs, we developed a Perl script to select only mCs with read coverages ≥2×, a FDR < 0.05, and that appeared in at least two replicates with the same methylation status. These reliable mCs were used for further analysis.

### Differentially methylated cytosines in genic regions

To analyze the methylation or nonmethylation statuses of all cytosines across all genic regions in the rice reference genome, including the promoters (2 kb upstream of each gene), gene bodies, and downstream regions (2 kb downstream of each gene), we retrieved the position information for each of these regions from the annotated rice reference genome in the MSU RGAP database, and used these positions for the following analysis.

To detect the differences in cytosine methylation patterns between the two coisogenic rice strains, the reliable mCs were first used to identify the DMCs between the experimental group and the control group for each strain. That is, if a given reliable mC was nonmethylated in the control group but methylated in the experimental group, the reliable mC was a DMC (HNT-induced methylation). Alternatively, if a given reliable mC was methylated in the control group but nonmethylated in the experimental group, the reliable mC was also a DMC (HNT-induced demethylation). Second, the methylation patterns of the DMCs were further compared between the two coisogenic rice strains. If a DMC was methylated or demethylated due to HNT in the heat-tolerant strain, but the methylation status of this DMC was reversed in the heat-sensitive strain, this DMC was considered differentially methylated between the heat-tolerant and heat-sensitive coisogenic rice strains. Third, the DMCs were classified into three types based on the difference in methylation patterns between the two coisogenic rice strains (Fig. 1b): in type I DMCs, the DMC in the heat-tolerant strain was methylated by HNT, but the corresponding cytosine in the heat-sensitive strain was either unchanged or demethylated by HNT; in type II DMCs, the DMC in the heat-tolerant strain was demethylated by HNT, but the corresponding cytosine in the heat-sensitive strain was either unchanged or methylated after HNT exposure; and in type III DMCs, the DMC in the heat-tolerant strain was unchanged after HNT exposure, but the corresponding cytosine in the heat-sensitive strain was either methylated or demethylated by HNT.

### Differentially methylated regions between the two coisogenic rice strains

As the efficient initiation of gene transcription requires a nonmethylated (methylation-free) DNA region downstream and/or upstream of the gene [23], we identified DMRs that included at least 15 successive DMCs that were consistently methylated or demethylated by HNTs, and where the methylation patterns of these DMCs differed between the heat-tolerant and heat-sensitive coisogenic rice strains. Specifically, we required that the DNA region be < 100 bp, with a GC content > 30%; that > 60% of all cytosines in the region were DMCs; and that at least 15 successive cytosines were either Type I, II, or III DMCs. We identified DMRs using a script written in awk (https://github.com/onetrueawk/awk). Genes with DMRs in the promoter, downstream, or gene body region were considered HNT-induced DMGs; DMGs were also classified as type I, II, or III based on the methylation patterns of the DMCs in the DMR.

### Validation of the WGBS data using BSP

To validate the WGBS data, we randomly selected six DMRs for BSP [54]. Genomic DNA samples from all TC, TT, SC, and ST replicates were first bisulfite-converted using EZ DNA Methylation-Gold kits. The bisulfite-converted DNA samples were then PCR amplified as previously described using bisulfite-specific primers (Additional file 1: Table S4), which were designed using Methyl Primer Express v1.0 (Applied Biosystems, USA). The PCR products were separated using 1% agarose gel electrophoresis. Target DNA fragments were then extracted and gel-purified using QIAEX II Gel Extraction Kits (Qiagen, Germany). The purified DNA was ligated with the pGEM-T Easy vector (Promega, USA) and cloned in *DH5α* cells (Takara, Japan). For each sample, 10 positive clones were selected and sequenced individually using Sanger sequencing.

### Functional annotation of the DMGs

To predict the molecular functions of the DMGs, we first searched for the gene IDs in the MSU RGAP database; DMGs not identified in the MSU RGAP were then searched against the UniProt database. The DMGs not successfully annotated against UniProt were searched against previously published studies. Finally, DMGs were classified based on their annotated molecular functions.

### Pathway enrichment

To identify the pathways overrepresented by DMGs, the MSU RGAP gene IDs for the DMGs were converted into Rice Annotation Project Database (RAP-DB, https://rapdb.dna.affrc.go.jp/tools/converter/run) gene IDs using the ID convert tool in the RAP-DB. The converted gene IDs were then searched against the KEGG database to identify the KEGG pathways enriched in the DMGs. We searched for unmatched DMGs or homologys in the literature, and then constructed a composite regulatory network based on the functional, biological, and pathway terms enriched in the DMGs.

### Validation of the relationships among methylation, gene expression, and heat tolerance in rice

To investigate relationships among cytosine methylation in the gene promoter region, gene expression, and heat tolerance, we used six rice germplasms: three heat-tolerant (Huazhan, Qiyinzhan, and Simiao) and three heat-sensitive (Labelle, Koshihikari, and OM997) [69]. Using these plant materials, we analyzed the relationship among the cytosine methylation rate in the region upstream of *PPR* (1577–1495 bp upstream to the TSS), *PPR* gene expression, and heat tolerance.

The six rice germplasms were cultivated and temperature treated as described above. Grain weights and differences in grain weight between the heat-tolerant and heat-sensitive strains were also calculated as described above. The methylated cytosine sites in the promoter region of the *PPR* gene were identified using the BSP method, with forward primer 5′-TATTGGGATTGGAGTGTAGGAT-3′ and reverse primer 5′-CCATARATTAATCATTTAARCACTAAC-3′. For each sample, 30 positive clones were sequenced in triplicate. We quantified the expression of the target gene (*PPR*) using semi-quantitative reverse transcriptase (RT)-PCR with *Actin1* as the control gene, as described previously [70]. The forward primer (5′-TGCTCACAAGGTGAAGTTGTCT-3′) and the reverse primer (5′-GGCAGTGGAATACTGGAACGA-3′) used for semi-quantitative RT-PCR, which spanned one intron, were designed with Primer-BLAST (https://www.ncbi.nlm.nih.gov/tools/primer-blast/). The semi-quantitative RT-PCRs were performed using the following cycling conditions: initial denaturation at 95 °C for 5 min; followed by 30 or 27 amplification cycles (30 for *PPR,* 27 for *Actin1*) of denaturation at 94 °C for 30 s, annealing at 60 °C for 30 s, and elongation at 72 °C for 30 s; and a final extension at 72 °C for 5 min. The PCR products were visualized using 2% agarose gels and imaged using the BioRad Gel DocTM XR+ imager (BioRad, Hercules, CA). Three repeats were performed.

## Supplementary information

**Additional file 1: Table S1.** Summary of the whole-genome bisulfite sequencing data. **Table S2.** Number of genome-wide covered cytosines and methylated cytosines. **Table S3.** Gene function and the methylation patterns of the DMCs in the gene DMRs. **Table S4.** Primers used for bisulfite-sequencing PCR. **Figure S1.** Positions of the cytosines and differentially methylated regions in the target genes. **Figure S2.** Methylation or demethylation ratio of cytosines for six selected target DMRs. **Figure S3.** The activity levels of SOD (a), POD (b), and CAT (c), as well as endogenous ABA concentrations (d) in the heat-tolerant rice germplasms (Huazhan, Qiyinzhan, and Simiao) and the heat-sensitive rice germplasms (Labelle, Koshihikari, and OM997).

**Additional file 2: Figure S4.** is the original full length gel image for Labelle, and **Figure S5** for Koshihikari, **Figure S6** for OM997, **Figure S7** for Huazhan, **Figure S8** for Qiyinzhan, **Figure S9** for Simiao in Fig. 6c. The DNA Maker is 250 bp marker, and the bands from top to bottom are 500 bp, 400 bp, 300 bp, 250 bp, 200 bp, 150 bp, 100 bp and 50 bp. The size of amplified gene *Actin1* and *PPR* were 155 bp and 188 bp, respectively.

## Data Availability

The datasets generated during this study are available in the Genome Sequence Archive at the Beijing Institute of Genomics (BIG) Data Center, Chinese Academy of Sciences, under accession number CRA002257 (http://bigd.big.ac.cn/gsa/s/287hg5j0).

## Abbreviations

BSP: Bisulfite-sequencing PCR
DMC: Differentially methylated cytosine
DMG: Differentially methylated gene
DMR: Differentially methylated region
HNT: High nighttime temperature
KEGG: Kyoto Encyclopedia of Genes and Genomes
mC: methylated cytosine
MSU RGAP: Michigan State University Rice Genome Annotation Project
RAP-DB: Rice Annotation Project Database
WGBS: Whole-genome bisulfite sequencing
