# Supplementary material for: Cytosine methylations in the promoter regions of genes involved in the cellular oxidation equilibrium pathways affect rice heat tolerance
Source: BMC Genomics. 2020 Aug 14;21:560. doi: 10.1186/s12864-020-06975-3 (PMC7430847; doi:10.1186/s12864-020-06975-3)

**Additional file 1**

**Supplementary Tables**

**Table S1:** Summary of the whole-genome bisulﬁte sequencing data. TC1, TC2, and TC3 are the three biological repeats for the heat-tolerant rice strain under control conditions, while TT1, TT2, and TT3 represent the heat-tolerant rice strain under experimental conditions. Similarly, SC1, SC2, and SC3 are the three biological repeats under control conditions, while ST1, ST2, and ST3 represent the heat-sensitive strain under experimental conditions.

| **Sample** | **Number of raw reads (millions)** | **Number of clean reads (millions, % of raw reads)** | **Unique best-mapped reads** | | **Bisulfite conversion rate**  **(%)** |
| --- | --- | --- | --- | --- | --- |
|  |  |  | **Number (millions, % of clean reads)** | **Genome coverage**  **(%)** |  |
| TC1 | 61.85 | 54.35 (87.9) | 24.49 (45.1) | 73.8 | 99.5 |
| TC2 | 70.97 | 63.11 (88.9) | 29.39 (46.6) | 75.2 | 99.5 |
| TC3 | 45.83 | 45.25 (98.7) | 16.76 (37.0) | 68.6 | 99.6 |
| TT1 | 55.32 | 49.96 (90.3) | 24.17 (48.4) | 73.7 | 99.5 |
| TT2 | 57.76 | 49.95 (86.5) | 24.70 (49.4) | 74.2 | 99.4 |
| TT3 | 60.72 | 51.82 (85.3) | 25.64 (49.5) | 74.5 | 99.4 |
| SC1 | 52.18 | 44.13 (84.6) | 21.54 (48.8) | 72.5 | 99.4 |
| SC2 | 47.22 | 43.70 (92.5) | 23.32 (53.4) | 73.7 | 99.5 |
| SC3 | 65.69 | 56.29 (85.7) | 26.95 (47.9) | 74.5 | 99.4 |
| ST1 | 56.83 | 43.76 (77.0) | 21.21 (48.5) | 73.2 | 99.4 |
| ST2 | 58.81 | 47.03 (80.0) | 23.15 (49.2) | 73.3 | 99.4 |
| ST3 | 56.07 | 46.20 (82.4) | 23.27 (50.4) | 73.8 | 99.4 |
| Total | 689.26 | 595.55 (86.4) | 284.60 (47.8) | – | – |

**Table S2:** Number of genome-wide covered cytosines and methylated cytosines. mC, methylated cytosine; TC and TT represent the heat-tolerant rice strain under control and experimental conditions, respectively; TC1–3 and TT1–3 represent biological replicates. SC and ST represent the heat-sensitive rice strain under control and experimental conditions, respectively; SC1–3 and ST1–3 represent biological replicates. CG, CHG, and CHH represent three different cytosine contexts.

| **Sample** | | **Covered cytosines (millions)** | | | | **Total number of mCs**  **(%)** | **Reliable mCs (million)** | | | |
| --- | --- | --- | --- | --- | --- | --- | --- | --- | --- | --- |
|  |  | **Total** | **Three context types (%)** | | |  | **Total** | **Three context types (%)** | | |
|  |  |  | **CG** | **CHG** | **CHH** |  |  | **CG** | **CHG** | **CHH** |
|  |  |  |  |  |  |  |  |  |  |  |
| TC | TC1 | 120.34 | 21.66  (18.0) | 20.42  (17.0) | 78.26  (65.0) | 21.31  (17.7) | 17.53 | 8.76  (50.0) | 4.58  (26.1) | 4.19  (23.9) |
|  | TC2 | 122.64 | 22.10  (18.0) | 20.78  (16.9) | 79.75  (65.0) | 22.61  (18.4) |  |  |  |  |
|  | TC3 | 111.94 | 20.07  (17.9) | 19.09  (17.1) | 72.77  (65.0) | 18.18  (16.2) |  |  |  |  |
|  |  |  |  |  |  |  |  |  |  |  |
| TT | TT1 | 120.14 | 21.60  (18.0) | 20.39  (17.0) | 78.14  (65.0) | 21.65  (18.0) | 17.65 | 8.78  (49.8) | 4.60  (26.1) | 4.27  (24.2) |
|  | TT2 | 121.06 | 21.86  (18.1) | 20.56  (17.0) | 78.65  (65.0) | 21.34  (17.6) |  |  |  |  |
|  | TT3 | 121.45 | 21.97  (18.1) | 20.63  (17.0) | 78.84  (64.9) | 22.04  (18.1) |  |  |  |  |
|  |  |  |  |  |  |  |  |  |  |  |
| SC | SC1 | 118.15 | 21.23  (18.0) | 20.09  (17.0) | 76.83  (65.0) | 21.23  (18.0) | 17.70 | 8.64  (48.8) | 4.61  (26.1) | 4.45  (25.1) |
|  | SC2 | 120.19 | 21.8  (18.1) | 20.45  (17.0) | 77.94  (64.8) | 21.39  (17.8) |  |  |  |  |
|  | SC3 | 121.56 | 22.03  (18.1) | 20.66  (17.0) | 78.88  (64.9) | 22.39  (18.4) |  |  |  |  |
|  |  |  |  |  |  |  |  |  |  |  |
| ST | ST1 | 119.38 | 21.64  (18.1) | 20.35  (17.0) | 77.39  (64.8) | 19.97  (16.7) | 16.85 | 8.59  (51.0) | 4.42  (26.2) | 3.84  (22.8) |
|  | ST2 | 119.57 | 21.7  (18.2) | 20.4  (17.1) | 77.47  (64.8) | 19.86  (16.6) |  |  |  |  |
|  | ST3 | 120.27 | 21.67  (18.0) | 20.44  (17.0) | 78.17  (65.0) | 19.96  (16.6) |  |  |  |  |
|  | |  |  |  |  |  |  |  |  |  |
| Total | | 1,436.68 | 259.32  (18.0) | 244.27  (17.0) | 933.10  (64.9) | 251.95  (17.5) | – | – | – | – |

**Table S3:** Gene function and the different methylation patterns of the DMCs in the gene DMRs. DMC, differentially methylated cytosines; DMR, differentially methylated region; TSS, transcription start site; TTS, transcription termination site; Dns, downstream gene region; Ups, upstream gene region; Gby, gene body; HNT, high nighttime temperature. I indicates a type I DMC, where the DMC in the heat-tolerant strain was methylated by HNT, but the corresponding cytosine in the heat-sensitive strain was either unchanged or demethylated after HNT exposure; II indicates a type II DMC, where the DMC in the heat-tolerant strain was demethylated by HNT, but the corresponding cytosine in the heat-sensitive strain was either unchanged or methylated after HNT exposure; III indicates type III DMC, where the DMC in the heat-tolerant strain was unchanged after HNT exposure, but the corresponding cytosine in the heat-sensitive strain was either methylated or demethylated by HNT.

| **Gene ID** | **Functional annotation** | **DMR position in TSS/TTS** | **Total cytosine number in DMR** | **DMCs number in DMR** | **Methylation type of DMCs** |
| --- | --- | --- | --- | --- | --- |
| **Transcription regulation** | |  |  |  |  |
| LOC_Os07g09540 | Transposon protein, CACTA, En/Spm sub-class | Dns_983-1042 | 22 | 18 | II |
| LOC_Os12g29230 | Transposon protein, CACTA, En/Spm sub-class | Ups_1623-1572 | 25 | 18 | I |
| LOC_Os10g20320 | Transposon protein, CACTA, En/Spm sub-class | Ups_1636-1557 | 31 | 19 | III |
| LOC_Os10g42460 | Retrotransposon protein, Ty3-gypsy subclass | Ups_1544-1497 | 21 | 17 | I |
| LOC_Os07g41530 | Retrotransposon protein, Ty3-gypsy subclass | Dns_855-910 | 25 | 17 | I |
| LOC_Os11g35730 | Retrotransposon protein, Ty1-copia subclass | Ups_1290-1221 | 35 | 22 | II |
| LOC_Os09g13780 | Unclassified retrotransposon protein | Ups_1024-972 | 21 | 17 | I |
| LOC_Os12g14900 | Unclassified retrotransposon protein | Dns_1848-1935 | 31 | 23 | III |
| LOC_Os09g15160 | Unclassified retrotransposon protein | Ups_1129-1088 | 26 | 19 | III |
| LOC_Os12g42750 | Unclassified transposon protein | Dns_633-679 | 41 | 25 | III |
| LOC_Os01g48810 | Transcription initiation factor TFIID subunit 11 | Dns_358-399 | 16 | 16 | II |
| LOC_Os07g07020 | Mediator of RNA polymerase II transcription subunit 31 | Dns_2-60 | 18 | 18 | II |
| LOC_Os08g33150 | MYB family transcription factor | Dns_1748-1799 | 22 | 18 | III |
| LOC_Os02g52780 | bZIP transcription factor | Dns_651-728 | 25 | 20 | I |
| LOC_Os11g47900 | GRAS family transcription factor containing protein | Dns_264-341 | 27 | 20 | II |
| LOC_Os04g11830 | TCP family transcription factor, sequence-specific DNA binding activity | Dns_1892-1965 | 26 | 19 | I |
| LOC_Os11g03110 | GRAS family transcription factor domain containing protein | Ups_456-392 | 26 | 18 | III |
| LOC_Os12g41030 | AP2 domain containing protein | Dns_683-739 | 22 | 19 | III |
| LOC_Os11g24850 | Ankyrin repeat domain containing protein | Dns_1246-1303 | 26 | 20 | I |
| LOC_Os07g28900 | PPR repeat domain containing protein | Ups_1577-1495 | 30 | 21 | II |
| LOC_Os12g39990 | Dof zinc finger domain containing protein | Dns_214-259 | 19 | 16 | I |
| LOC_Os01g68860 | Zinc finger C-x8-C-x5-C-x3-H type family protein | Dns_1253-1318 | 29 | 18 | III |
| LOC_Os03g41390 | ZOS3-15 - C2H2 zinc finger protein, sequence-specific DNA binding activity | Dns_1669-1737 | 30 | 18 | III |
| LOC_Os03g63810 | WRKY80, sequence-specific DNA binding transcription factor activity | Dns_1347-1439 | 32 | 24 | I |
| LOC_Os02g11030 | 50S ribosomal protein L19, chloroplast precursor | Dns_1282-1334 | 22 | 16 | II |
| LOC_Os01g73770 | DREB protein, DNA-binding transcription factor activity | Dns_714-789 | 24 | 17 | I |
| LOC_Os09g03610 | Flowering time control protein FCA, RNA binding | Dns_1730-1800 | 26 | 17 | I |
| LOC_Os01g19694 | Homeobox domain containing protein | Gby_2653-2745 | 36 | 22 | II |
| LOC_Os01g19490 | Pentatricopeptide | Dns_602-679 | 24 | 17 | III |
| LOC_Os03g32620 | Pentatricopeptide | Dns_563-635 | 27 | 19 | II |
| LOC_Os04g47690 | HMG1/2 | Ups_429-387 | 18 | 17 | II |
| LOC_Os04g24190 | Growth-regulating factor 11, transcription | Ups_351-287 | 22 | 17 | I |
| **Energy metabolism** | |  |  |  |  |
| LOC_Os02g05890 | Chloroplast proton-transporting ATP synthase complex assembly | Ups_1746-1684 | 21 | 18 | I |
| LOC_Os04g02030 | Rp1 | Dns_1903-1949 | 21 | 17 | II |
| LOC_Os12g06920 | NBS-LRR disease resistance protein, ADP binding | Dns_1061-1129 | 26 | 21 | I |
| LOC_Os01g49430 | DnaK (HSP70) family protein | Dns_81-162 | 30 | 23 | II |
| LOC_Os06g08310 | Plasma membrane ATPase, hydrolase activity | Ups_616-538 | 26 | 19 | III |
| LOC_Os01g49360 | FAD-binding and arabino-lactone oxidase domains containing protein | Ups_589-520 | 28 | 20 | III |
| LOC_Os04g35280 | Neutral/alkaline invertase | Ups_926-860 | 24 | 16 | I |
| LOC_Os10g05660 | Thaumatin, acting as endo‐β‐1,3‐glucanases on polymeric β‐1,3‐glucans | Ups_323-245 | 26 | 17 | III |
| LOC_Os04g02900 | Dehydrogenase E1 component domain containing protein, pyruvate dehydrogenase activity | Dns_1643-1692 | 42 | 27 | III |
| LOC_Os04g45580 | Kinesin motor domain containing protein, ATPase activity | Ups_518-433 | 27 | 17 | II |
| **Transport** | |  |  |  |  |
| LOC_Os03g18560 | DUF538 domain containing protein | Ups_365-283 | 27 | 17 | II |
| LOC_Os05g50160 | DUF617 domain containing protein | Dns_725-818 | 30 | 18 | III |
| LOC_Os07g40460 | Prefoldin, protein binding | Dns_1286-1314 | 17 | 16 | I |
| LOC_Os03g14080 | Transmembrane amino acid transporter protein, transporter activity | Ups_487-434 | 24 | 20 | I |
| LOC_Os01g73810 | ATCHX15, transporter activity | Ups_340-302 | 20 | 16 | III |
| LOC_Os01g55610 | Peptide transporter PTR2 | Dns_121-169 | 23 | 17 | II |
| LOC_Os04g32880 | CBS domain containing membrane protein | Ups_328-284 | 24 | 17 | III |
| LOC_Os01g12130 | Nodulin MtN3 family protein | Dns_310-394 | 31 | 21 | III |
| **Signal transduction** | |  |  |  |  |
| LOC_Os01g62370 | ENTH domain containing protein | Dns_172-226 | 17 | 16 | III |
| LOC_Os01g04230 | Inactive receptor kinase At2g26730 precursor, kinase activity | Dns_886-946 | 24 | 21 | II |
| LOC_Os02g16610 | Harpin-induced protein 1 domain containing protein | Dns_1379-1439 | 21 | 17 | I |
| LOC_Os05g30750 | Anthranilate phosphoribosyltransferase | Ups_1919-1862 | 24 | 18 | III |
| LOC_Os04g47680 | Ser/Thr-rich protein T10 in DGCR region | Dns_213-306 | 31 | 21 | III |
| LOC_Os03g59390 | CAMK_CAMK_like | Dns_1097-1184 | 36 | 24 | III |
| LOC_Os04g30110 | Wall-associated receptor kinase 3 precursor | Ups_1279-1203 | 29 | 18 | I |
| LOC_Os08g03440 | Actin cytoskeleton | Ups_1778-1722 | 26 | 16 | I |
| **Metabolism** | |  |  |  |  |
| LOC_Os03g18390 | Cysteine-type peptidase activity | Dns_1316-1352 | 16 | 16 | I |
| LOC_Os08g09750 | OsFBL44-F-box domain and LRR containing protein | Ups_326-264 | 20 | 16 | III |
| LOC_Os10g25340 | GDSL-like lipase/acylhydrolase, hydrolase activity | Ups_458-386 | 33 | 24 | II |
| LOC_Os05g06660 | OsSCP26-Putative Serine Carboxypeptidase homologue, hydrolase activity | Ups_711-639 | 23 | 16 | I |
| LOC_Os04g35114 | Receptor-like kinase, kinase activity | Dns_672-768 | 41 | 26 | II |
| LOC_Os04g48020 | 3-Hexulose-6-phosphate isomerase | Ups_609-521 | 29 | 18 | III |
| **Oxidation** | |  |  |  |  |
| LOC_Os08g38720 | Cytochrome c oxidase assembly protein COX15 | Ups_260-209 | 22 | 18 | III |
| LOC_Os08g37874 | 2-Nitropropane dioxygenase family | Dns_65-114 | 22 | 16 | II |
| LOC_Os01g49120 | MATE efflux family protein | Ups_1495-1443 | 29 | 18 | I |
| **Function unknown** | |  |  |  |  |
| LOC_Os07g15100 | Hypothetical protein | Ups_1822-1745 | 32 | 28 | III |
| LOC_Os08g36820 | Expressed protein | Dns_686-747 | 23 | 20 | II |
| LOC_Os12g18070 | Expressed protein | Ups_255-186 | 22 | 19 | I |
| LOC_Os08g33790 | Hypothetical protein | Dns_463-518 | 20 | 17 | III |
| LOC_Os09g04120 | Expressed protein | Ups_1848-1795 | 22 | 18 | I |
| LOC_Os01g59940 | Expressed protein | Ups_556-508 | 20 | 16 | III |
| LOC_Os09g38650 | Expressed protein | Ups_1482-1418 | 23 | 18 | III |
| LOC_Os08g05040 | Expressed protein | Dns_51-114 | 24 | 18 | III |
| LOC_Os04g19310 | Expressed protein | Dns_163-260 | 31 | 23 | I |
| LOC_Os06g47290 | Growth regulator related protein | Dns_822-879 | 23 | 17 | I |
| LOC_Os11g19170 | Hypothetical protein | Ups_767-698 | 23 | 17 | I |
| LOC_Os05g15230 | Plant domain containing protein | Dns_1521-1552 | 22 | 16 | II |
| LOC_Os05g48770 | Expressed protein | Dns_760-823 | 22 | 16 | I |
| LOC_Os03g41030 | Expressed protein | Ups_1249-1189 | 22 | 16 | III |
| LOC_Os06g08730 | Expressed protein | Ups_503-435 | 22 | 16 | III |
| LOC_Os05g42428 | Expressed protein | Dns_296-388 | 33 | 24 | III |
| LOC_Os08g07490 | Expressed protein | Dns_1281-1345 | 24 | 17 | II |
| LOC_Os03g46940 | Expressed protein | Ups_1125-1059 | 24 | 17 | II |
| LOC_Os01g51470 | Expressed protein | Ups_1366-1322 | 23 | 16 | III |
| LOC_Os04g53420 | Expressed protein | Ups_1859-1810 | 23 | 16 | III |
| LOC_Os07g36950 | Expressed protein | Ups_1641-1562 | 26 | 18 | III |
| LOC_Os09g36720 | Expressed protein | Dns_246-313 | 25 | 17 | I |
| LOC_Os08g31590 | Expressed protein | Dns_1786-1844 | 27 | 18 | I |
| LOC_Os01g35240 | Hypothetical protein | Dns_644-721 | 30 | 20 | I |
| LOC_Os09g15360 | Hypothetical protein | Dns_1752-1810 | 24 | 16 | III |
| LOC_Os08g35120 | Expressed protein | Ups_1813-1728 | 29 | 19 | II |
| LOC_Os06g51300 | Expressed protein | Ups_602-530 | 26 | 17 | II |
| LOC_Os04g28390 | Expressed protein | Dns_1358-1443 | 39 | 25 | III |
| LOC_Os01g16590 | Expressed protein | Ups_1975-1908 | 25 | 16 | II |
| LOC_Os01g55190 | Expressed protein | Ups_521-431 | 29 | 18 | III |
| LOC_Os01g08750 | Expressed protein | Ups_1991-1903 | 28 | 17 | II |
| LOC_Os12g42650 | Pollen preferential protein | Ups_838-803 | 28 | 17 | III |

**Table S4**: Primers used for bisulfite-sequencing PCR. Primers were designed to amplify the regions differentially methylated between the heat-tolerant and the heat-sensitive coisogenic rice strains. F and R indicate the forward and reverse primers, respectively; Y indicates a pyrimidine (cytosine or thymine).

|  | **Target gene** | **Region** | **Primer** | **Product size (bp)** |
| --- | --- | --- | --- | --- |
| DMR-1 | LOC_Os03g63810 | Dns_1347-1439 | F: GTTGGTAATATTAATYGGGATTAA | 275 |
|  |  |  | R: AAAAATTTTTTTTCTTCTTCTTTTTAA |  |
|  |  |  |  |  |
| DMR-2 | LOC_Os04g02030 | Dns_1903-1949 | F: AGTTAGGTTAGGTTAATATTTTTGGT | 320 |
|  |  |  | R: AAATAATCTTCCTCTCTTATCTATCC |  |
|  |  |  |  |  |
| DMR-3 | LOC_Os07g40460 | Dns_1286-1314 | F: GGAAAATGAGTTGTTATTTTTTTTA | 353 |
|  |  |  | R: CCACTTTCTCATAAAAAACTCATA |  |
|  |  |  |  |  |
| DMR-4 | LOC_Os08g09750 | Ups_326-264 | F: GAGATTGGGATTAAAAATTAAATTTT | 321 |
|  |  |  | R: ATCCTAACCCACCCACTTATAT |  |
|  |  |  |  |  |
| DMR-5 | LOC_Os08g03440 | Ups_1778-1722 | F: TTTTGTGTGGTGATTTTTTAATT | 399 |
|  |  |  | R: CCAATTATATTCCATCAAATTCATA |  |
|  |  |  |  |  |
| DMR-6 | LOC_Os08g36820 | Dns_686-747 | F: TTAAAAAYGGTGGAAATTTATT | 302 |
|  |  |  | R: ATACCCTACCAATTCATTAACAA |  |

**Supplementary Figures**

**Figure S1**: Positions of the cytosines and differentially methylated regions in the target genes. HTS and HSS indicate the heat-tolerant strain and heat-sensitive strain, respectively. Dots along the horizontal axes represent cytosines. Areas boxed in red, blue, and green dashed lines are the differentially methylated regions (DMRs): regions that are consistently methylated or demethylated by HNT in the successive cytosines genome-wide but showed a different methylation pattern between the heat-tolerant and heat-sensitive rice strains. Type I (in red dashed lines): the DMC in the heat-tolerant strain was methylated by HNT, but the corresponding cytosine in the heat-sensitive strain was either unchanged or demethylated after HNT exposure; Type II (in blue dashed line): the DMC in the heat-tolerant strain was demethylated by HNT, but the corresponding cytosine in the heat-sensitive strain was either unchanged or methylated after HNT exposure; Type III (in green dashed line): the DMC in the heat-tolerant strain was unchanged after HNT exposure, but the corresponding cytosine in the heat-sensitive strain was either methylated or demethylated by HNT. Vertical bars extending above the horizontal axes represent cytosines methylated by HNT, while vertical bars extending below the horizontal axes represent cytosines demethylated by HNT.

**
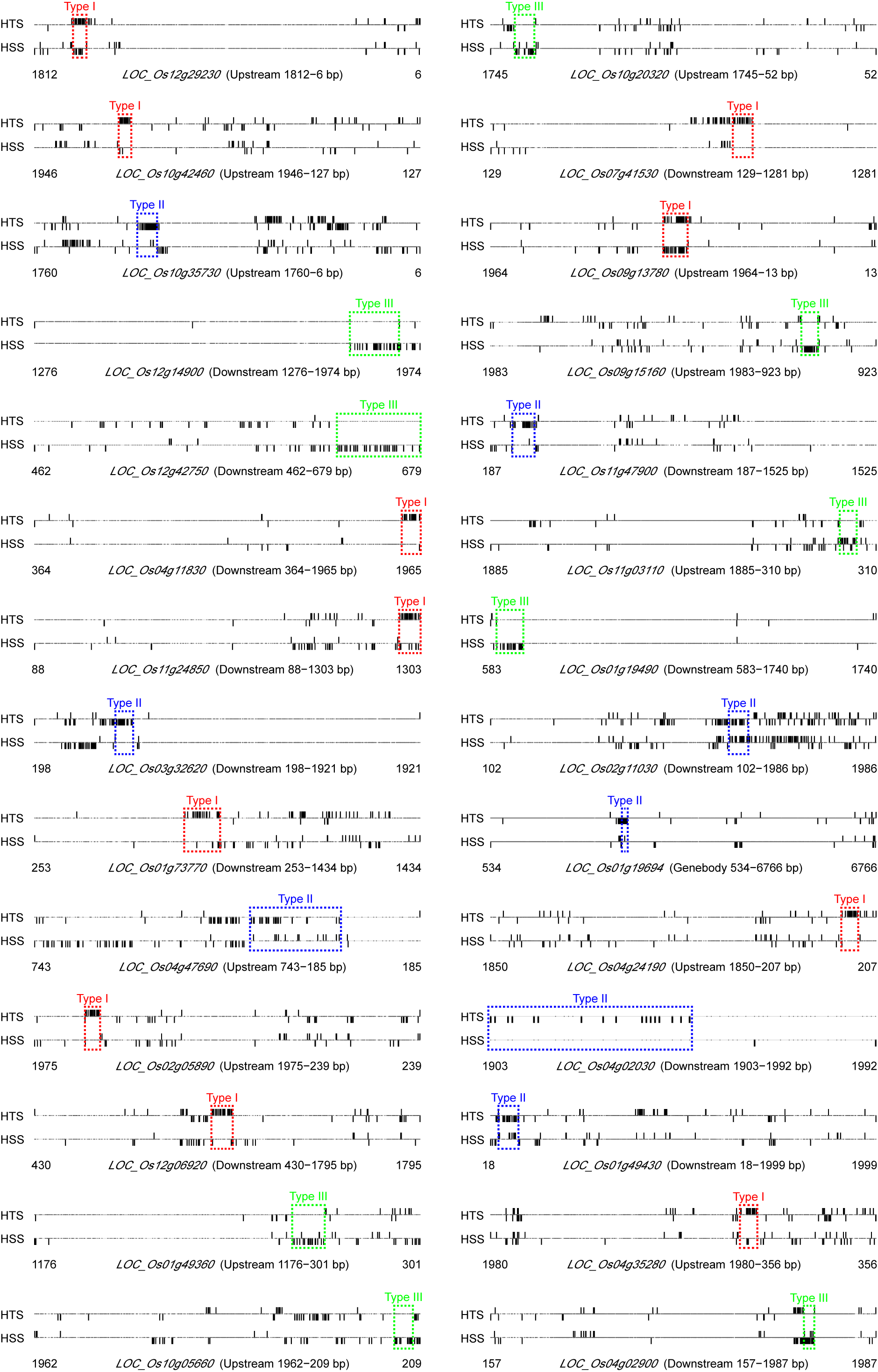
**

**
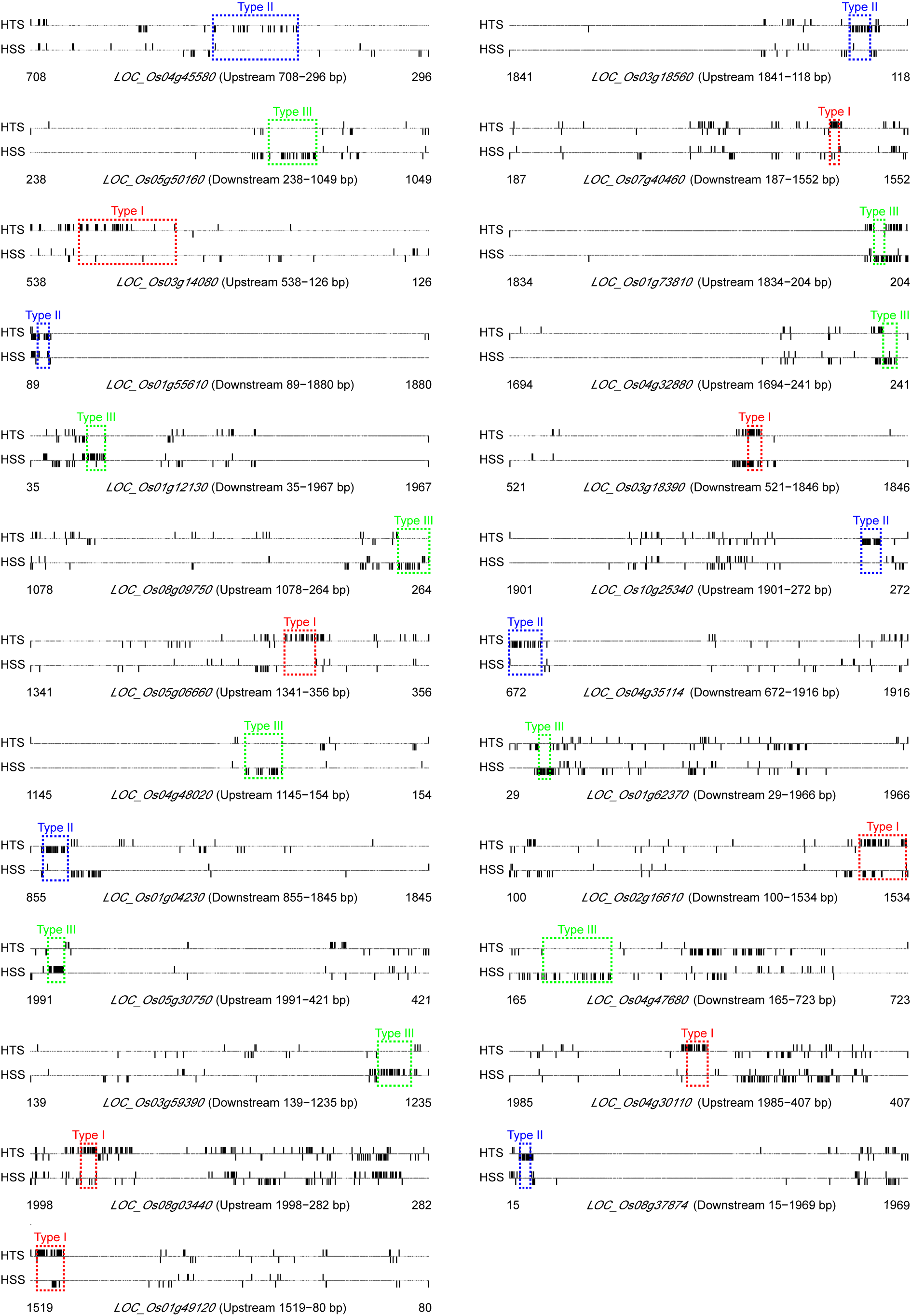
**

**Figure S2**: Methylation or demethylation ratio of cytosines for six selected target DMRs. DMR indicates differentially methylated region; WGBS indicates whole-genome bisulfite sequencing; BSP indicates bisulfite-sequencing PCR; TC and TT represent the heat-tolerant rice strain under control and experimental conditions, respectively; SC and ST represent the heat-sensitive rice strain under control and experimental conditions, respectively. The nucleotide sequences of the DMRs are plotted along the horizontal axes. Black pillars represent the methylation rate generated from WGBS, and purple pillars represent the methylation rate generated from BSP.

**
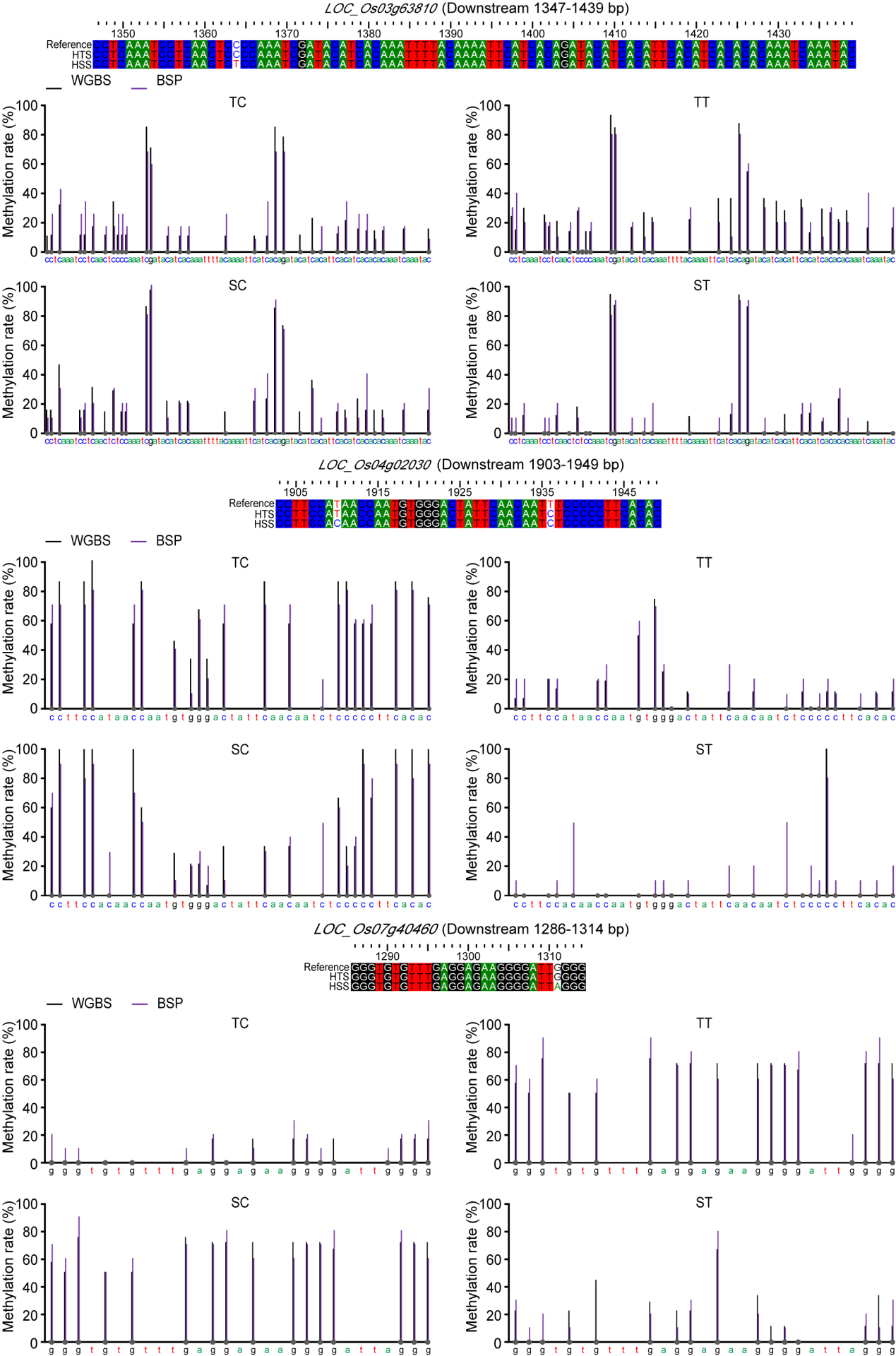
**

**
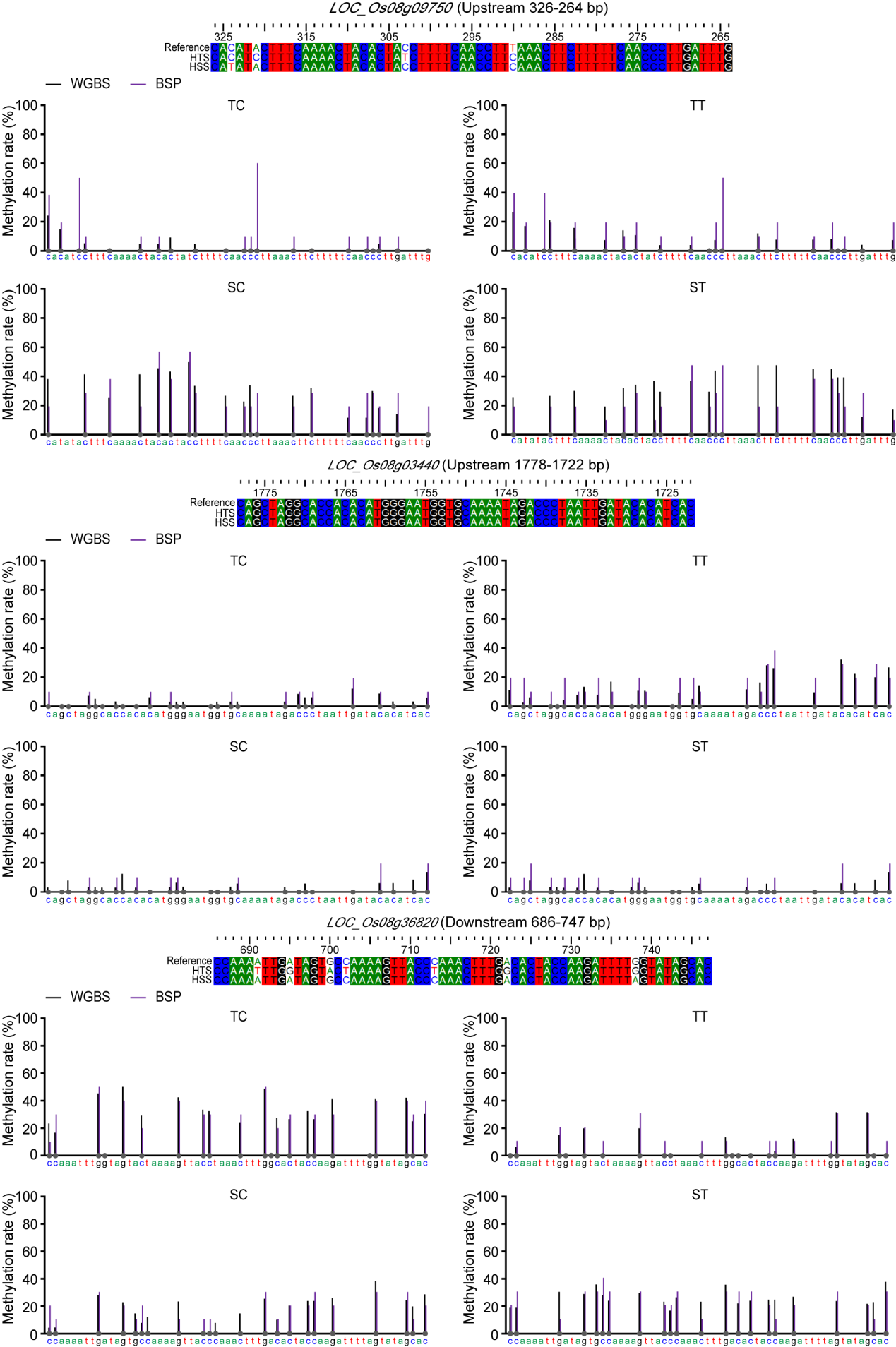
**

**Figure S3**. The activity levels of superoxide dismutase (SOD) (a), peroxidase (POD) (b), and catalase (CAT) (c), as well as endogenous abscisic acid (ABA) concentrations (d) in the heat-tolerant rice germplasms (Huazhan, Qiyinzhan, and Simiao) and the heat-sensitive rice germplasms (Labelle, Koshihikari, and OM997). The activity levels of the ROS-related enzymes (SOD, POD, and CAT) and the endogenous ABA concentrations in the rice grains were detected using specific assay kits (Suzhou Comin Co., Ltd, Suzhou, China), following the manufacturer's instructions. ABA levels were detected using ultra-high-performance liquid chromatography coupled to tandem mass spectrometry (UHPLC-MS/MS). First, rice grains (100 mg per sample) were frozen in liquid nitrogen and ground into a powder. Then, 50 mg of powder per sample was mixed with 20 μl of internal standard solution (Sigma-Aldrich, Steinheim, Canada) and 1 ml precooled 50% ACN/H_2_O (v/v). Each mixture was vortexed and sonicated in an ice-water bath twice. Next, samples were centrifuged and the supernatants (800 μl per sample) were purified using solid phase extraction cartridges (Waters Corporation, Milford, MA, USA). The purified samples were loaded into an EXIONLC System (AB Sciex) equipped with a Waters ACQUITY UPLC CSH C18 column (150 × 2.1 mm, 1.7 μm, Waters) for UHPLC separation. Mobile phase A was 0.01% formic acid in ultrapure water, and mobile phase B was 0.01% formic acid in acetonitrile. The column temperature was set to 50℃, the auto-sampler temperature was set to 4℃, and the injection volume was 1 μl. The separated ABA was qualified using multiple reaction monitoring (MRM) via an AB SCIEX 6500 QTRAP + triple quadrupole mass spectrometer (AB Sciex) equipped with an electrospray ionization (ESI) interface. The MRM parameters were optimized using flow injection analysis by injecting the standard solutions of the individual analytes into the API source of the mass spectrometer. The ion source parameters were as follows: curtain gas, 40 psi; ionspray voltage, ±4500 V; temperature, 475℃; ion source gas 1, 30 psi; and ion source gas 2, 30 psi. AB SCIEX Analyst Work Station Software (1.6.3 AB SCIEX) and Sciex OS Version 1.4.0 were used for MRM data acquisition and processing. The results are shown as means of three biological replicates. In response HNT stress, the activity levels of SOD, POD, and CAT were elevated in both the heat-tolerant and the heat-sensitive rice germplasms. However, these levels were higher in the heat-tolerant germplasms as compared to the heat-sensitive germplasms (Fig. S3). Endogenous ABA concentrations in the heat-tolerant germplasms were similar to those in the heat-sensitive rice germplasms under normal conditions, and HNT stress significantly increased ABA concentration both strains. However, the increase in ABA content was noticeably greater in the heat-tolerant germplasms.


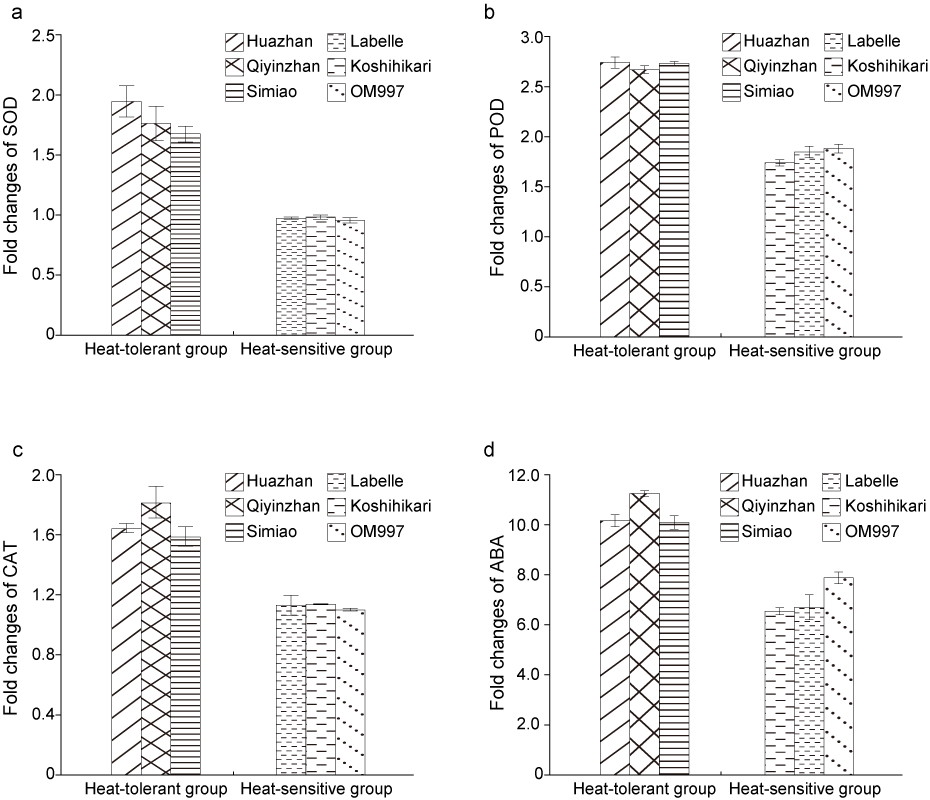

Supplement: Supplementary file 1 — Additional file 1: Table S1. Summary of the whole-genome bisulfite sequencing data. Table S2. Number of genome-wide covered cytosines and methylated cytosines. Table S3. Gene function and the methylation patterns of the DMCs in the gene DMRs. Table S4. Primers used for bisulfite-sequencing PCR. Figure S1. Positions of the cytosines and differentially methylated regions in the target genes. Figure S2. Methylation or demethylation ratio of cytosines for six selected target DMRs. Figure S3. The activity levels of SOD (a), POD (b), and CAT (c), as well as endogenous ABA concentrations (d) in the heat-tolerant rice germplasms (Huazhan, Qiyinzhan, and Simiao) and the heat-sensitive rice germplasms (Labelle, Koshihikari, and OM997). [file 12864_2020_6975_MOESM1_ESM.docx]
