# Supplementary material for: Cytosine methylations in the promoter regions of genes involved in the cellular oxidation equilibrium pathways affect rice heat tolerance
Source: BMC Genomics. 2020 Aug 14;21:560. doi: 10.1186/s12864-020-06975-3 (PMC7430847; doi:10.1186/s12864-020-06975-3)
Supplement: Supplementary file 2 — Additional file 2: Figure S4. is the original full length gel image for Labelle, and Figure S5 for Koshihikari, Figure S6 for OM997, Figure S7 for Huazhan, Figure S8 for Qiyinzhan, Figure S9 for Simiao in Fig. 6c. The DNA Maker is 250 bp marker, and the bands from top to bottom are 500 bp, 400 bp, 300 bp, 250 bp, 200 bp, 150 bp, 100 bp and 50 bp. The size of amplified gene Actin1 and PPR were 155 bp and 188 bp, respectively. [file 12864_2020_6975_MOESM2_ESM.doc]

**Additional file 2**

**
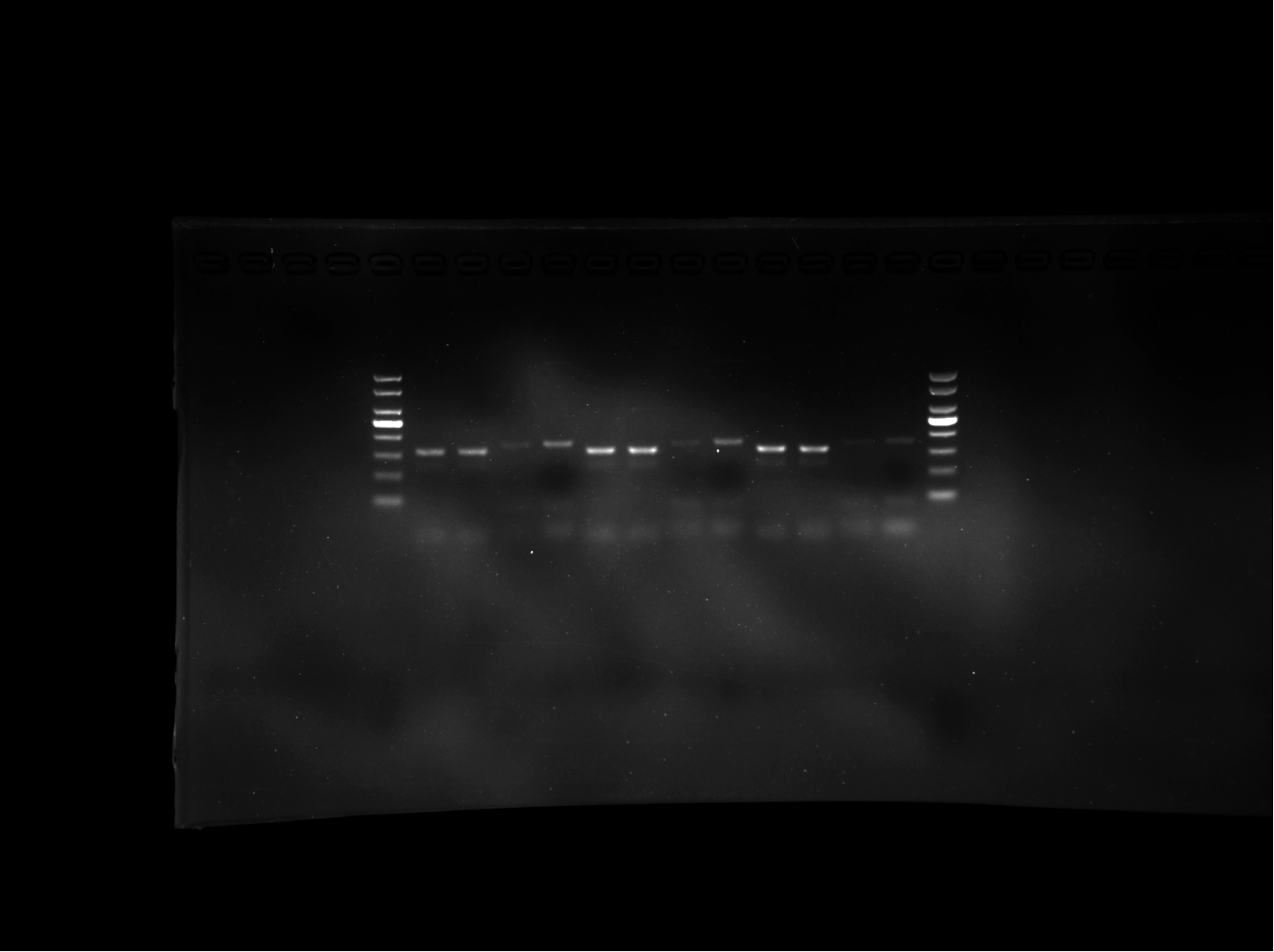
**

**Figure S4. Original gel image for Labelle in Fig. 6c:** The expression of gene *PPR* for the typical heat-sensitive rice germplasms Koshihikari under high nighttime temperature and normal temperature condition. The DNA Maker is 250 bp marker, and the bands from top to bottom are 500 bp, 400 bp, 300 bp, 250 bp, 200 bp, 150 bp, 100 bp and 50 bp. The size of amplified gene *Actin1* and *PPR* were 155 bp and 188 bp, respectively.

**
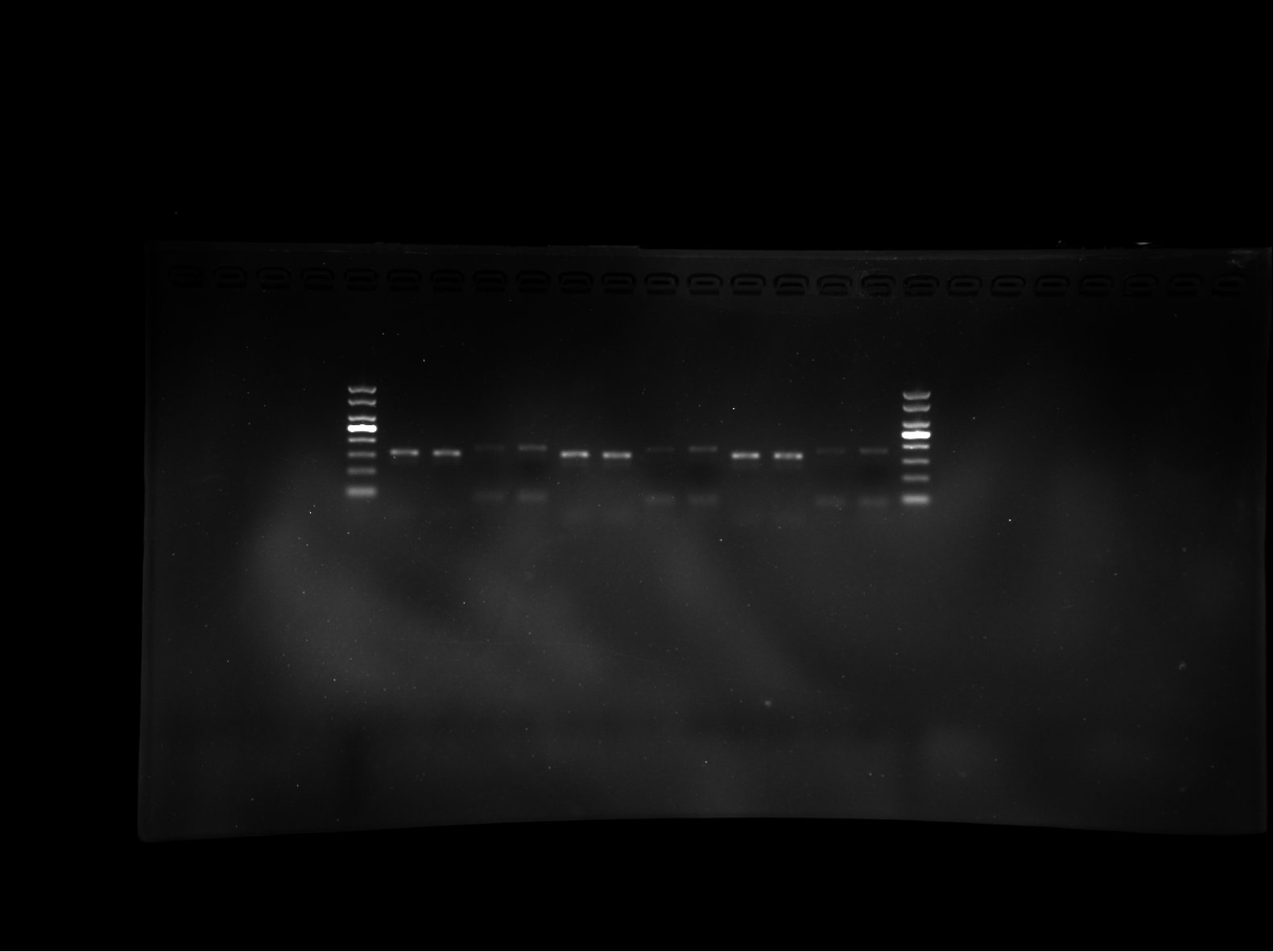
**

**Figure S5. Original gel image for Koshihikari in Fig. 6c:** The expression of gene *PPR* for the typical heat-sensitive rice germplasms Labelle under high nighttime temperature and normal temperature condition. The DNA Maker is 250 bp marker, and the bands from top to bottom are 500 bp, 400 bp, 300 bp, 250 bp, 200 bp, 150 bp, 100 bp and 50 bp. The size of amplified gene *Actin1* and *PPR* were 155 bp and 188 bp, respectively.

**
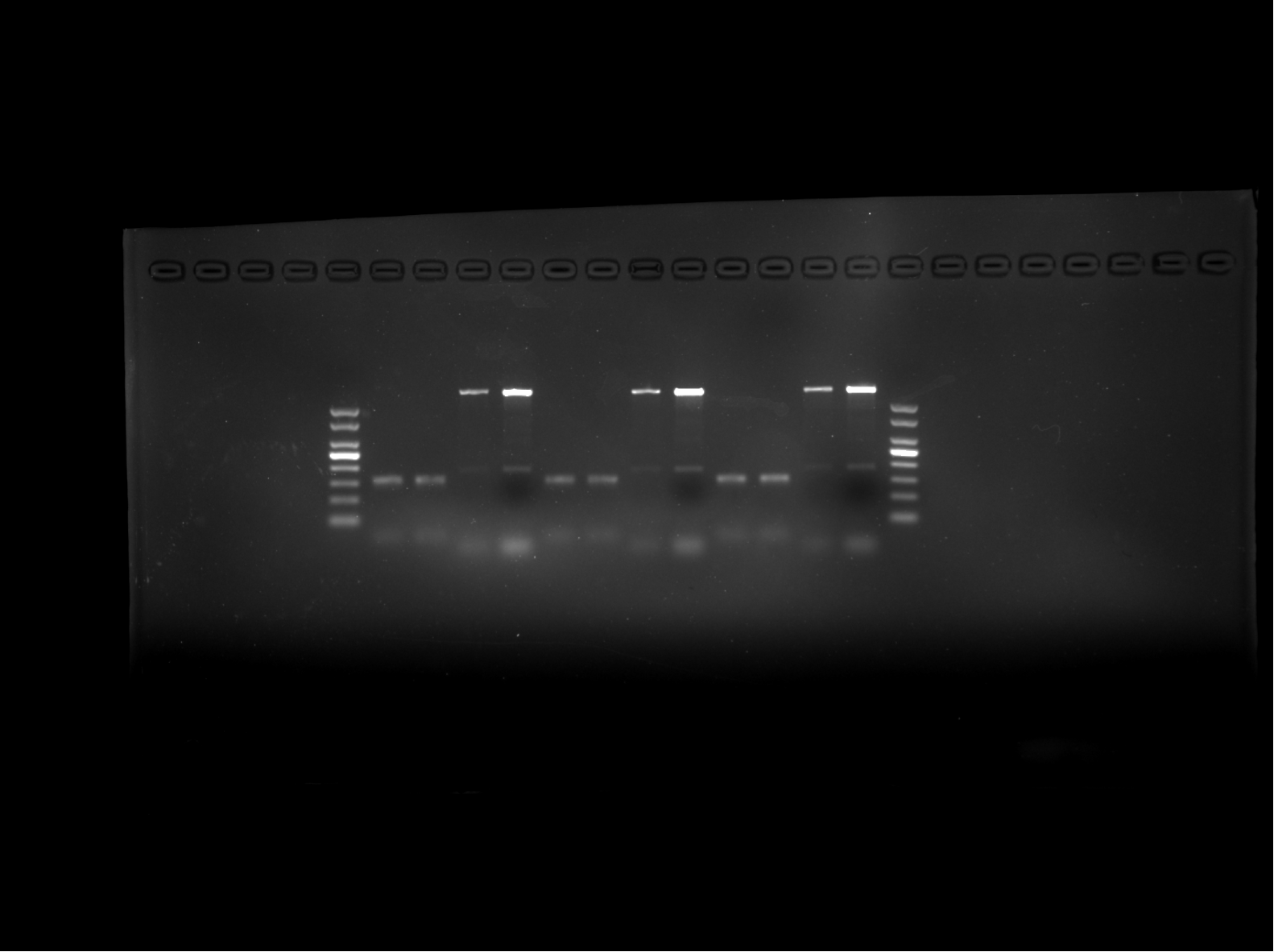
**

**Figure S6. Original gel image for OM997 in Fig. 6c:** The expression of gene *PPR* for the typical heat-sensitive rice germplasms OM997 under high nighttime temperature and normal temperature condition. The DNA Maker is 250 bp marker, and the bands from top to bottom are 500 bp, 400 bp, 300 bp, 250 bp, 200 bp, 150 bp, 100 bp and 50 bp. The size of amplified gene *Actin1* and *PPR* were 155 bp and 188 bp, respectively.

**
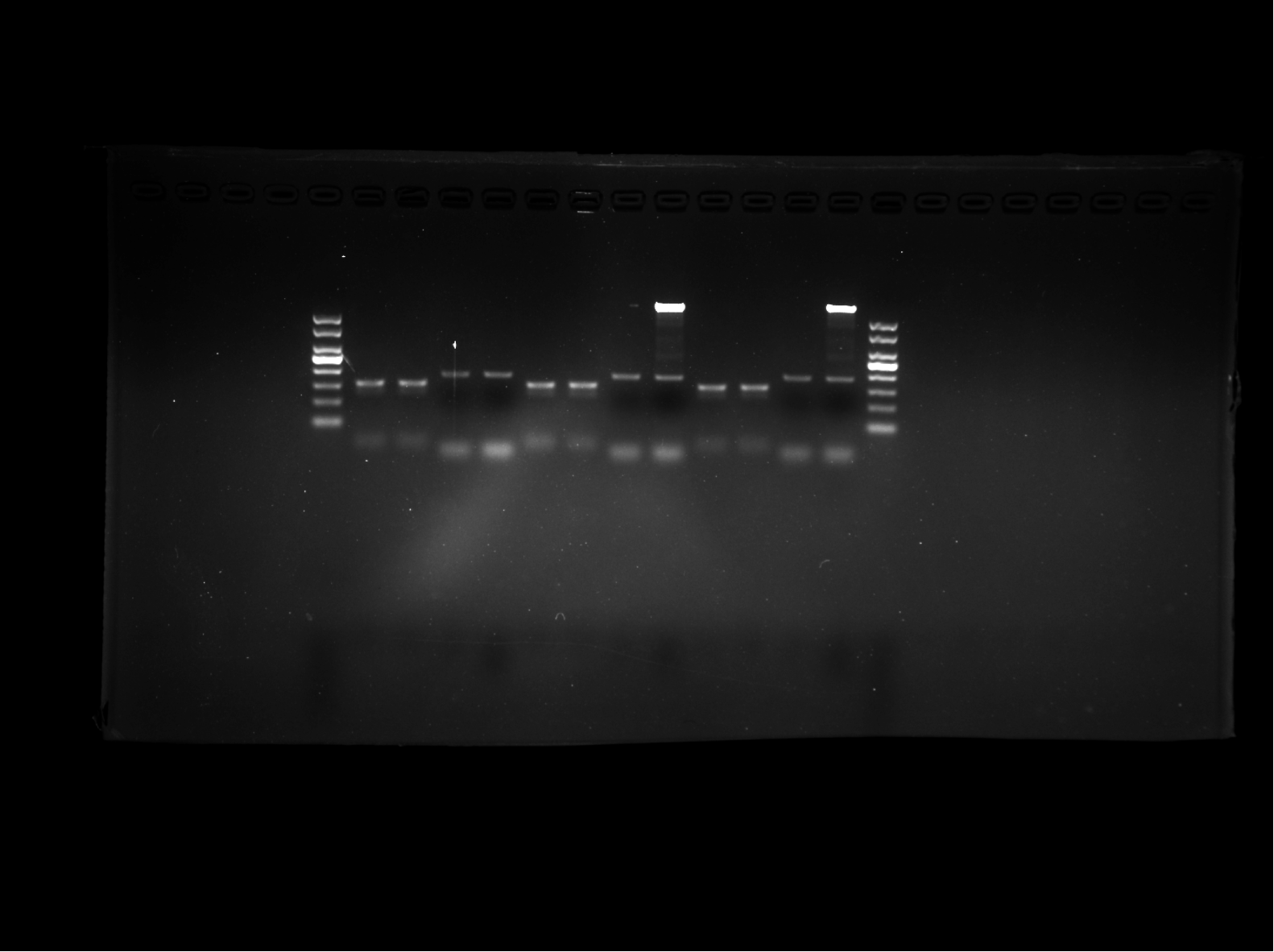
**

**Figure S7. Original gel image for Huazhan in Fig. 6c:** The expression of gene *PPR* for the typical heat-tolerant rice germplasms Huazhan under high nighttime temperature and normal temperature condition. The DNA Maker is 250 bp marker, and the bands from top to bottom are 500 bp, 400 bp, 300 bp, 250 bp, 200 bp, 150 bp, 100 bp and 50 bp. The size of amplified gene *Actin1* and *PPR* were 155 bp and 188 bp, respectively.

**
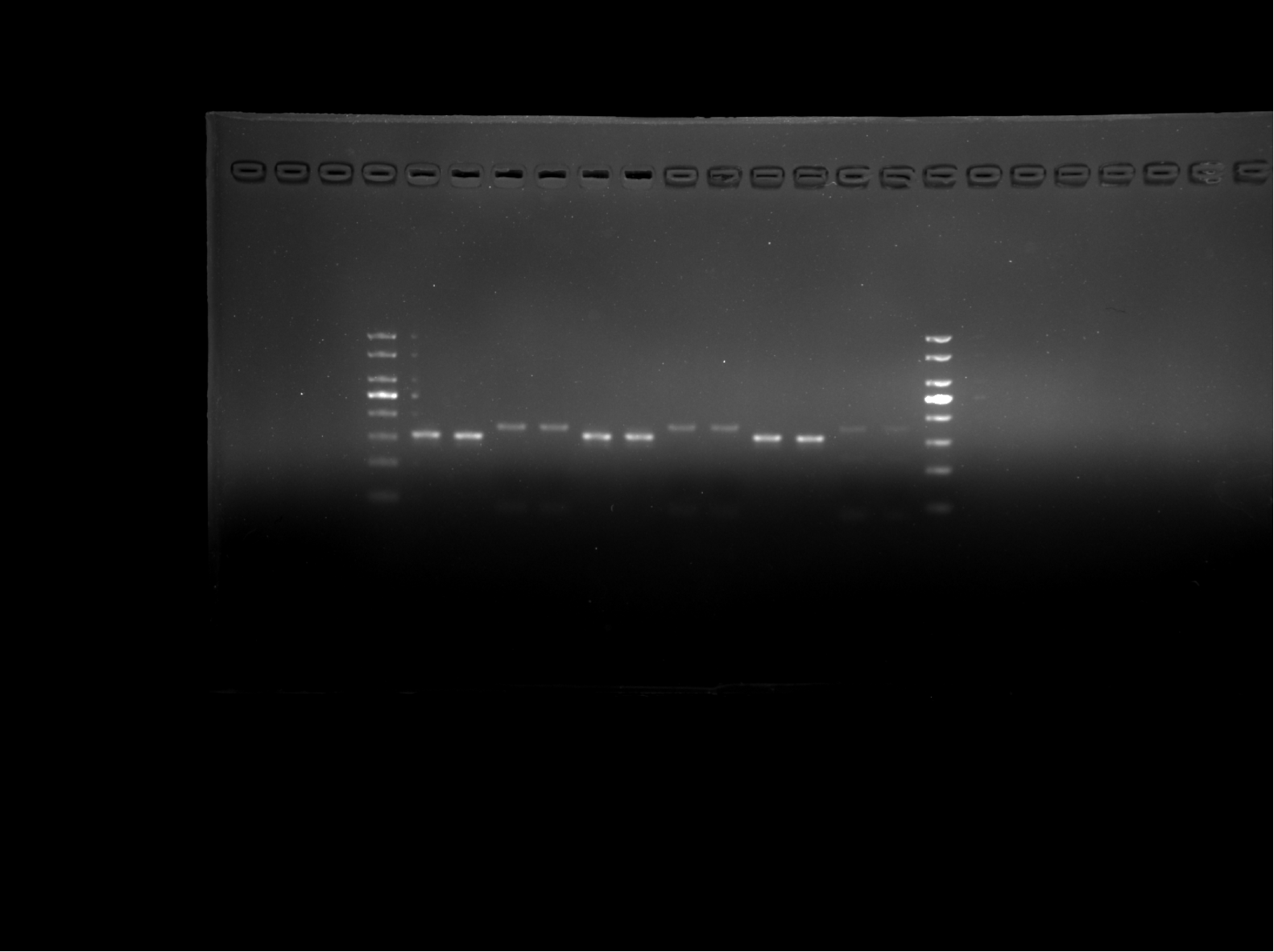
**

**Figure S8. Original gel image for Qiyinzhan in Fig. 6c:** The expression of gene *PPR* for the typical heat-tolerant rice germplasms Qiyinzhan under high nighttime temperature and normal temperature condition. The DNA Maker is 250 bp marker, and the bands from top to bottom are 500 bp, 400 bp, 300 bp, 250 bp, 200 bp, 150 bp, 100 bp and 50 bp. The size of amplified gene *Actin1* and *PPR* were 155 bp and 188 bp, respectively.

**
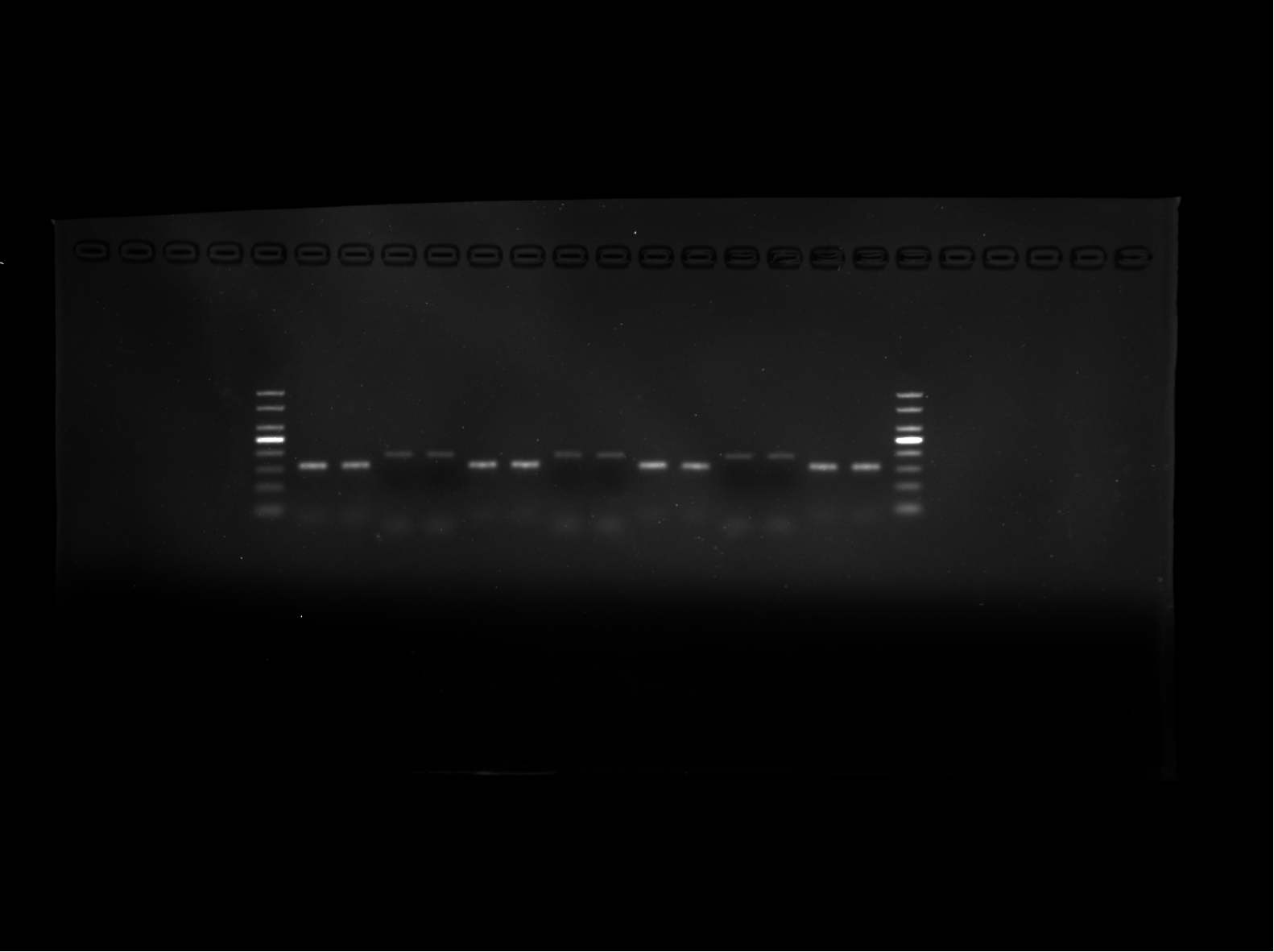
**

**Figure S9. Original gel image for Simiao in Fig. 6c:** The expression of gene *PPR* for the typical heat-tolerant rice germplasms Simiao under high nighttime temperature and normal temperature condition. The DNA Maker is 250 bp marker, and the bands from top to bottom are 500 bp, 400 bp, 300 bp, 250 bp, 200 bp, 150 bp, 100 bp and 50 bp. The size of amplified gene *Actin1* and *PPR* were 155 bp and 188 bp, respectively.
